# Identification of Drought Tolerant Mechanisms in Maize Seedlings Based on Transcriptome Analysis of Recombination Inbred Lines

**DOI:** 10.3389/fpls.2016.01080

**Published:** 2016-07-26

**Authors:** Haowei Min, Chengxuan Chen, Shaowei Wei, Xiaoling Shang, Meiyun Sun, Ran Xia, Xiangguo Liu, Dongyun Hao, Huabang Chen, Qi Xie

**Affiliations:** ^1^State Key Laboratory of Plant Genomics and National Center for Plant Gene Research, Institute of Genetics and Developmental Biology, Chinese Academy of SciencesBeijing, China; ^2^Argo-Biotechnology Research Institute, Jilin Academy of Agricultural SciencesChangchun, China

**Keywords:** transcriptomic profile, turgor homeostasis, detoxification signaling, drought stress, *Zea mays*

## Abstract

*Zea mays* is an important crop that is sensitive to drought stress, but survival rates and growth status remain strong in some drought-tolerant lines under stress conditions. Under drought conditions, many biological processes, such as photosynthesis, carbohydrate metabolism and energy metabolism, are suppressed, while little is known about how the transcripts of genes respond to drought stress in the genome-wide rang in the seedling stage. In our study, the transcriptome profiles of two maize recombination inbred lines (drought-tolerant RIL70 and drought-sensitive RIL93) were analyzed at different drought stages to elucidate the dynamic mechanisms underlying drought tolerance in maize seedlings during drought conditions. Different numbers of differentially expressed genes presented in the different stages of drought stress in the two RILs, for the numbers of RIL93 vs. RIL70 were: 9 vs. 358, 477 vs. 103, and 5207 vs. 152 respectively in DT1, DT2, and DT5. Gene Ontology enrichment analysis revealed that in the initial drought-stressed stage, the primary differentially expressed genes involved in cell wall biosynthesis and transmembrane transport biological processes were overrepresented in RIL70 compared to RIL93. On the contrary, differentially expressed genes profiles presented at 2 and 5 day-treatments, the primary differentially expressed genes involved in response to stress, protein folding, oxidation-reduction, photosynthesis and carbohydrate metabolism, were overrepresented in RIL93 compared to RIL70. In addition, the transcription of genes encoding key members of the cell cycle and cell division processes were blocked, but ABA- and programmed cell death-related processes responded positively in RIL93. In contrast, the expression of cell cycle genes, ABA- and programmed cell death-related genes was relatively stable in RIL70. The results we obtained supported the working hypothesis that signaling events associated with turgor homeostasis, as established by cell wall biosynthesis regulation- and aquaporin-related genes, responded early in RIL70, which led to more efficient detoxification signaling (response to stress, protein folding, oxidation-reduction) during drought stress. This energy saving response at the early stages of drought should facilitate more cell activity under stress conditions and result in drought tolerance in RIL70.

## Introduction

Drought is a primary abiotic constraint affecting crop production worldwide, owing to climate change, and shortages of freshwater associated with population growth (Hu and Xiong, 2014; Lobell et al., 2014). Upon drought stress treatment, a series of cellular processes involving morphological, physiological and biochemical changes to cope with dehydration, such as leaf rolling, stomatal closure, membrane stability, osmotic adjustment, antioxidant accumulation, reactive oxygen species (ROS) scavenging and transcription activation, are affected (Valliyodan and Nguyen, 2006; Jogaiah et al., 2013; Guerra et al., 2015). Many studies have been performed to understand the molecular mechanisms of drought stress, and some genes related to dehydrins (Kosova et al., 2014), transporters (Aharon et al., 2003; Li et al., 2015), and transcription factors (Hu et al., 2006; Nakashima et al., 2009), regarded as regulators influencing the drought tolerance of plants, were identified. Although, many molecular elements have been identified, the gene network of the drought stress response is still not fully elucidated (Zhu, 2002).

Maize (*Zea mays* L.), a primary food crop, exceeding rice, wheat and other cereals in importance since 2012 (Opitz et al., 2014), is sensitive to drought, and suffers significant decreases in corn production under drought stress in different regions (Todaka et al., 2015). The seeding stage of maize is especially sensitive to drought stress. In arid and semiarid regions such as North China, crops often undergo drought stress in spring and early summer, when water deficits threaten germination and seedling growth. Maize seedlings require less water than the later vegetative and reproductive stages, but water stress will influence their adaptation at the early crop establishment phase and their grain yield potential, due to premature flowering and a longer anthesis-silk interval (Maiti et al., 1996; Cao and Wj, 2004). To reveal the mechanism of how maize responds to drought at the seedling stage and to improve maize early crop establishment in regions where drought occurs during the early crop development phase are the major targets of maize drought-resistant breeding.

Cell turgor loss is arguably the best recognized classical indicator of plant water stress, with impacts on cellular structural integrity, metabolism, and whole-plant performance (McDowell, 2011). It was suggested that changes such as ceasing cell wall synthesis might be a mechanism by which plant cells withstand the changes in turgor pressure caused by osmotic stress during drought conditions (Tenhaken, 2014). Other reports revealed that some transmembrane proteins, such as AQPs, are likely to be of fundamental significance to all facets of plant growth and development, through regulating cell turgor pressure under osmotic stress induced by factors such as salinity and drought and influencing the cell–water status in plants (Forrest and Bhave, 2007).

Previous reports have shown that drought stress increases the accumulation of abscisic acid (ABA) in maize reproductive tissue, enhancing or depleting starch levels, decreasing endosperm cell division and potentially affecting many downstream processes after pollination (Zinselmeier et al., 1999; Setter and Flannigan, 2001; Parra, 2010). The influence of ABA on apoplastic sugar transport such as cell wall invertase and hexose transporters leading to pollen sterility in abiotic stressed rice was also observed (Oliver et al., 2007). However, no comparable effects of ABA on the seedling stage in maize have been reported.

The huge influence of drought stress on maize occurs at the very early period in seedling development and likely involves stress responses that affect various processes in the early seedlings. Although, there have been several reports of drought tolerance studies between different inbred lines at the seedling stage (Xinhai et al., 2004; Bin et al., 2007) that demonstrated the diversity of drought tolerance among different inbred lines, the mechanisms underlying this phenomenon and the genes involved in drought resistance at this early stage are still not well known. To date, few genome-wide approaches to this phenomenon have been reported. In the past, hybridization-based microarrays were established for acquiring gene expression profiles from maize tassels, ears, leaves and roots to analyze plant drought responses (Zhuang et al., 2007; Spollen et al., 2008; Marino et al., 2009; Zheng et al., 2010; Jogaiah et al., 2013). However, high-throughput transcript studies of drought-stressed maize seedlings have not been undertaken.

In this study, a population of 181 recombinant inbred lines (RILs) was obtained by self-crosses between the ph4CV and F9721 parental lines for six generations from the F1 generation. And the two parental lines for the RIL population, ph4CV showed drought-tolerant phenotype, and F9721 showed drought-sensitive phenotype under the same drought conditions (Figure S1). Including the two parental lines, the RIL population was exposed to drought conditions, and two extreme drought-responsive lines, the drought-tolerant line RIL70 and the drought-sensitive line RIL93, were identified. The results of deep RNA sequencing of the two drought responses revealed extremely different RILs, and the comparison of their transcriptomes in different drought stages is presented here. The data reveal the effects of drought stress on transcript abundance profiles from drought-sensitive and drought-tolerant lines under different drought conditions. The diversity of GO categories enriched in the dynamic process of drought stress indicates the complexity and the differences in the response between the two RILs. Quantitative PCR analysis demonstrated the validity of the RNA-Seq data, and could be used to clarify the phenomenon in the future.

## Results

### Phenotypic and physiological responses of the RILs to drought stress

**Figure 1:**
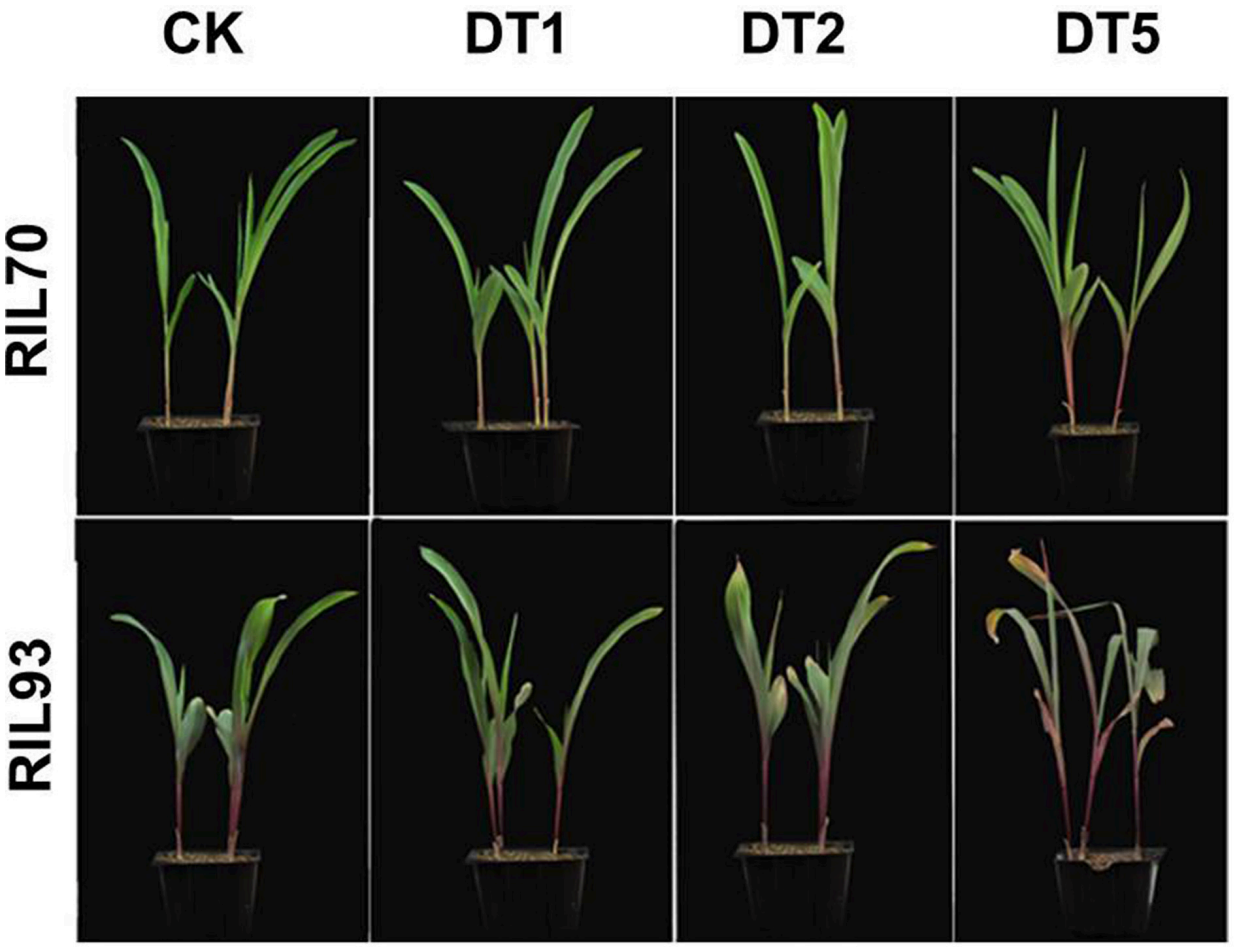
**The phenotype of RIL70 and RIL93 treated with drought at different (0, 1, 2, and 5) days**.

Two maize recombination inbred lines, RIL70 and RIL93, were chosen to study the drought response mechanism of maize in the 3-leaf seedling stage. Generally, the seedlings of RIL70 presented markedly higher growth vigor than RIL93 under drought stress (Figure 1, Figure S2). The relative water content (RWC) of both genotypes gradually decreased with prolonged drought stress, but the reductions were more severe in RIL93 (Figure S3), in which values decreased from 91.4 to 68.3% for the normal conditions to 5 days after drought stress.

**Figure 2:**
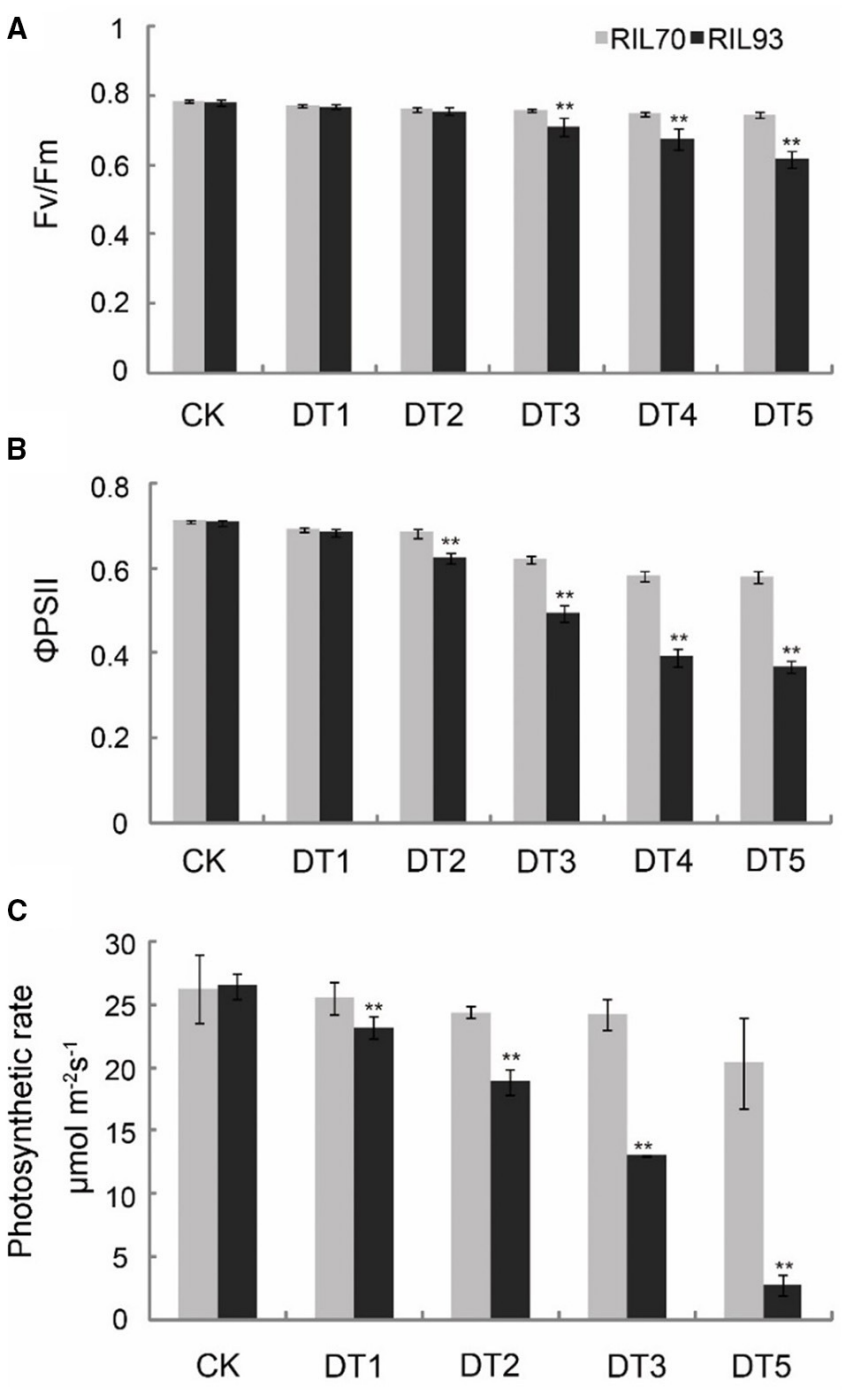
**Effect of drought stress on Fv/Fm, ΦPSII, and photosynthetic rate in leaves from RIL70 and RIL93**. Fv/Fm and ΦPSII were measured after treatment with drought for 0, 1, 2, 3, 4, and 5 days. Photosynthetic rates were measured after treatment with drought for 0, 1, 2, 3, and 5 days. Values are means ± *SD* of three measurements for each of four plants. Bars with two stars are significantly different at *p* = 0.01, respectively, according to a *t*-test.

In both genotypes, the potential efficiency of PSII photochemistry (Fv/Fm) was reduced with prolonged drought stress (Figure 2). Significant diversity was present at 3 days after drought stress treatment, when the Fv/Fm of RIL70 and RIL93 had decreased 4.2 and 9.1%, respectively. At 5 days after drought stress treatment, the Fv/Fm of RIL70 and RIL93 had decreased 4.9 and 21%, respectively (Figure 2A). The actual PSII efficiency (ΦPSII) decreased in both genotypes after drought stress. However, the reductions were greater in RIL93 (Figure 2B). In RIL70, ΦPSII decreased from 3 and 18.4%, and in RIL93, ΦPSII decreased from 3.3 to 48% from the initial stress condition to the 5 days after drought stress, respectively.

There were significant changes in the photosynthetic rate in both of the RILs as drought stress progressed, and photosynthesis in RIL93 was more intensely influenced under drought stress (Figure 2C). The photosynthetic rate in RIL93 was significantly inhibited from 2 days after drought ensued, and presented an aggravated tendency later in the drought process. The reduction in the photosynthetic rate of RIL93 was 28.8 and 89.6% at 2 and 5 days after drought stress, respectively, and the reduction in the photosynthetic rate of RIL70 was 7.6 and 22.8% at the corresponding stages. Furthermore, there was significant diversity in growth and, thus, a distinct difference in survival rates of the seedlings (Figure S2). The seedlings of RIL70 under severe drought stress recovered within 4 days after re-watering; however, the seedlings of RIL93 were more sensitive to drought stress. The survival rate of RIL70 was 95% compared to 2.5% in RIL93.

RNA isolated from seedlings that had been subjected to drought stress induced by treatment with or without water for 1–5 days was used for the RNA-Seq transcriptome analysis.

### Differential expression analysis of the maize seedling drought transcriptome

**Figure 3:**
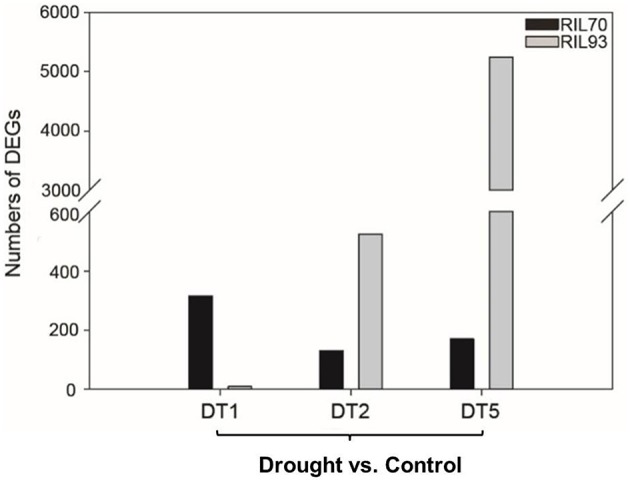
**Numbers of DEGs distributed in different RILs affected by different drought stress conditions**.

In this study, RNA-seq analysis was performed to obtain a comprehensive view of the maize gene expression profile under various conditions. Each condition is represented by two biological replicates per RIL, resulting in 4 pairs of samples and 16 samples in total. After filtering, a total of 1.8 billion clean reads were generated, with nearly 58 million reads on average from each sample. More than 85% of reads were at the Q30 level (an error probability less than 0.1%; Table S1). And the Table S2 were listed to show the mapping rate of every sample. Reads per kilobases of exon per million reads mapped (RPKM) was used to measure the transcript abundance of each gene. To measure the gene expression level for two replicates each sample, we calculated the Pearson correlation between samples and show in a heatmap (Figure S4). Each *R*^2^ between two replicates is more than 95%. In the absence of drought, 2104 genes showed differential expression levels when comparing RIL70 to RIL93 (Figure S5). However, the differentially expressed genes (DEGs) between them were 3274, 3851, and 6193 in different drought stress conditions (1, 2, and 5 days after drought stress). For the individual seedling, as shown in Figure 3, 314 genes were induced in RIL70, with 9 in RIL93 at 1 day after drought stress. Among these DEGs, 276 genes and 4 genes were up-regulated in drought-tolerant and -sensitive seedlings, respectively. There were only 131 genes with increased or decreased transcript abundance in RIL70, while the corresponding number in RIL93 was 523 at 2 days after drought stress. Among these DEGs, 82 genes and 305 genes were up-regulated in drought-tolerant and -sensitive seedlings, respectively. The expression of 170 genes was induced in RIL70 and a greater number of genes had different expression profiles in RIL93 at 5 days after drought stress. Among these DEGs, 46 genes and 2685 genes were up-regulated in drought-tolerant and -sensitive seedlings, respectively. All of these DEGs were selected for further analysis.

### The dynamic analysis of functional GO categories between the RIL lines under drought stress

**Figure 4:**
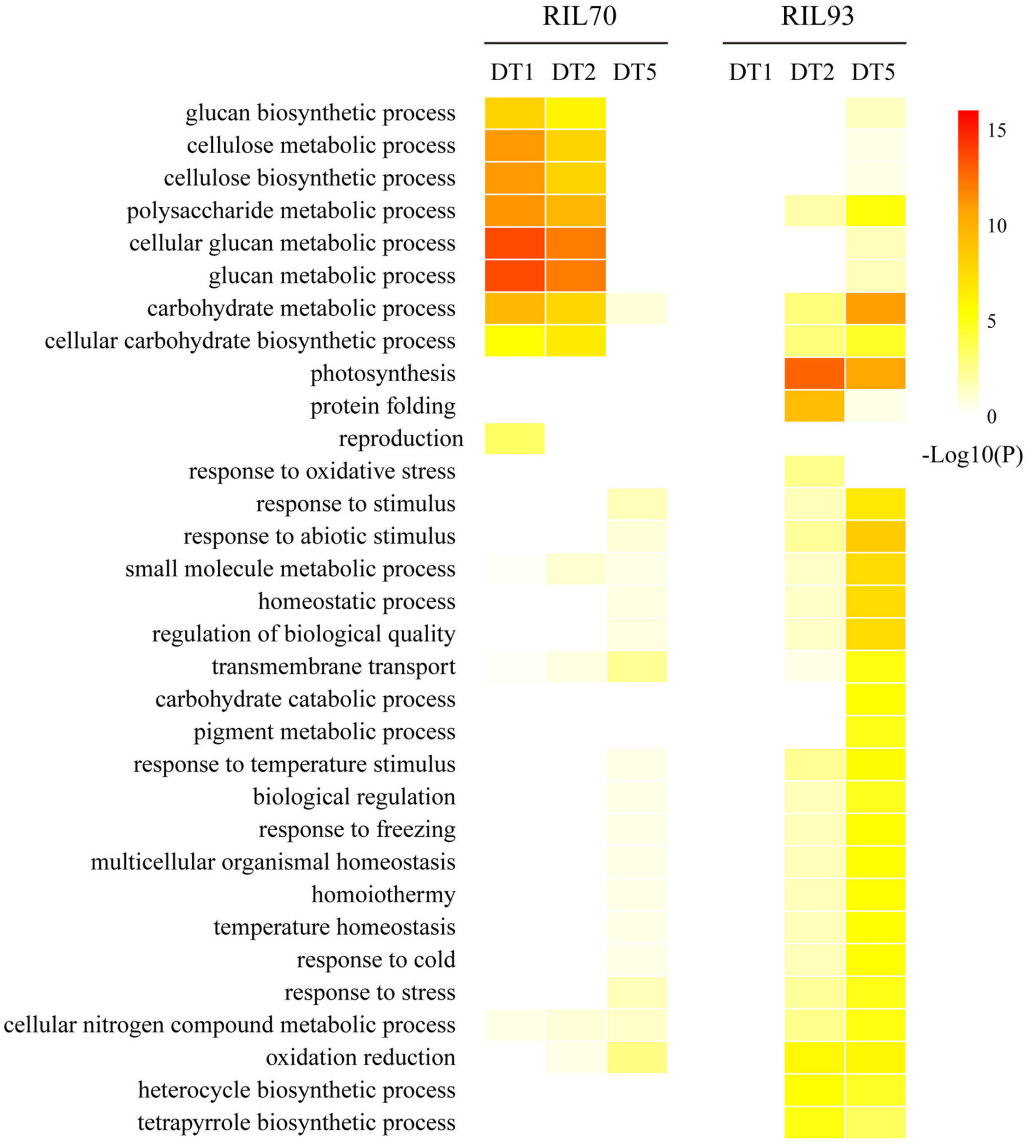
**The heat-map of GO enrichment of biological processes on differences between different time-points in the dynamic process of drought treatment in RIL70 relative to RIL93**. GO enrichment were determined by AgriGO.

To investigate the differences in drought-response mechanisms in the two RIL lines, GO enrichment was used to evaluate an overview of the dynamic profiles of functional categories by the enrichment level of DEGs. From the heat-map representation (Figure 4), it is evident that the drought-tolerant line responded earlier to drought stress than the drought-sensitive line at the initial stage (1 day after drought stress), while it was less affected by drought stress than the drought-sensitive line at the following stages (2 and 5 days after drought stress). Several distinct over-representation patterns of gene expression specific to one of the two lines in the dynamic progression of drought stress are shown in Figure 4. GO categories corresponding to many aspects of cell wall metabolism (“cellulose biosynthesis process” and “cellulose metabolic process”) and “transmembrane transport” were overrepresented at 1 and/or 2 days of drought stress in RIL70. On the contrary, these categories were overrepresented only after 5 days of drought stress in RIL93. The categories such as “response to stress” and “response to abiotic stress” were overrepresented in the 2 days after drought stress in RIL93, but only overrepresented in the 5 days after drought stress in RIL70, and the enrichment degree was much higher in RIL93 compared to RIL70. In the case of these GO categories, “protein folding,” “oxidation-reduction,” and “photosynthesis” were absent in the initial drought stress stage, and overrepresented at 2 and 5 days of drought stress in both lines. The enrichment of these categories in RIL93 was also much higher than in RIL70. Overall, this analysis revealed functional GO overrepresentation of DEGs related to cell wall organization, transmembrane transport, response to stress, protein folding, and photosynthesis, which might be involved in the drought response process of the two RILs. A select few of these categories were chosen for detailed examination.

### The regulation of cell wall related genes was closely related to the tolerance to drought stress

**Figure 5:**
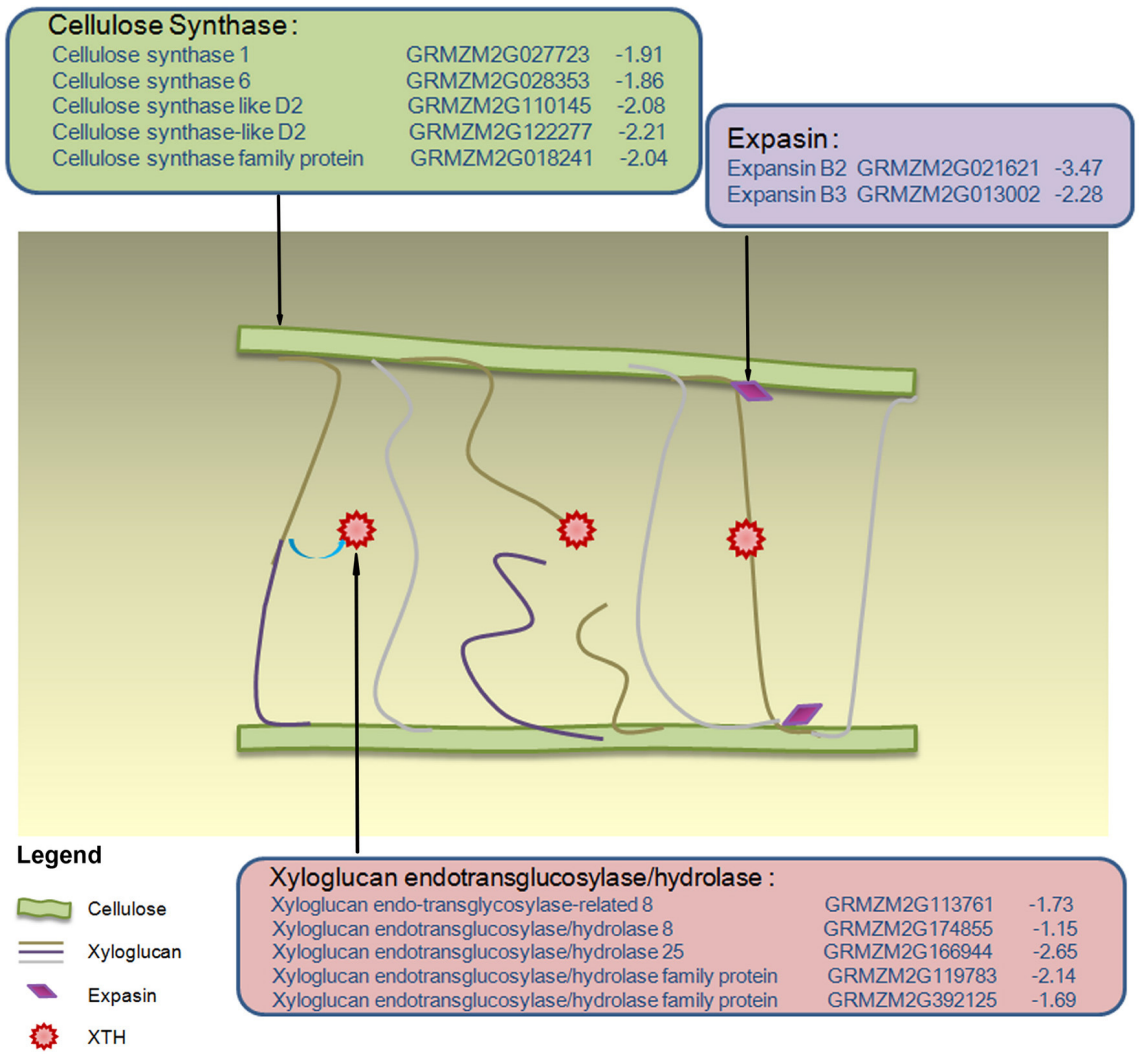
**Model of the changed expression profiles of cell wall growth genes in drought-stressed RIL70**. Different colored-box represent different groups of gene products involved in cell wall biosynthesis 1 and 2 days after drought stress. Within each colored-box, the expression data for drought-stressed RIL70 are listed in the form annotation, maize gene identifier, and log2fold change. The values in blue indicate the down-regulated log2fold change in expression in drought-stressed RIL70.

The dynamic GO enrichment results revealed that several groups of genes involved in cell wall biogenesis presented obvious expression changes specific in RIL70 but absented in RIL93 at 1 and 2 days after drought stress. And these groups of DEGs appeared and presented more obvious enrichment in RIL93 at 5 days after drought stress. As shown in Table S3, different numbers of DEGs distributed in the GO category “cellulose biosynthesis process” showed diverse expression profiles between RIL70 and RIL93 during drought stress. Among the DEGs at 1 day after drought stress, eleven genes encoding cellulose synthase, nine genes annotated as “*expansin*” and “*expansin-like*,” five *xyloglucan endo-transglycosylase* (*XTH*) genes, an *XTH-related* gene and a chitinase protein-encoding gene showed decreased expression in RIL70, while none of these genes were differentially expressed under drought stress in RIL93 (Table S3). Similarly, among the DEGs at 2 days after drought stress, six genes encoding cellulose synthase, three genes annotated as “*expansin*,” and six *xyloglucan endo-transglycosylase* (*XTH*) genes showed decreased expression in RIL70, but no gene expression changes were evident in RIL93. These data suggested that the synthesis of the cell wall might be hampered in the drought- tolerant line, but was not affected in the drought-sensitive line at 1 and 2 days after drought stress treatment. And the model in the Figure 5 also depicted the changed expression profiles of these DEGs presented both in the 1 and 2 days after drought-stress in RIL70. At 5 days after drought stress, most changes in DEGs involved in the cell wall biosynthesis process were in the drought-sensitive line. Among these genes, five genes encoding cellulose synthase showed increased expression, while three genes showed decreased expression in RIL93. Five genes annotated as “*expansin*” showed increased expression, while four genes showed decreased expression in RIL93. Two *pectin methylesterase inhibitor* genes showed decreased expression, while another *pectin methylesterase inhibitor* gene showed increased expression in RIL93. Two genes encoding a Pectin lyase-like superfamily protein showed increased transcript abundance in RIL93. Other genes involved in cell wall organization or biogenesis were increased, except for the *fucosyltransferase 1* gene. No genes belonging to these terms showed changed expression, except for a reversibly glycosylated polypeptide 2-encoding gene in RIL70. These data suggest that the regulation of cell wall biogenesis may contribute to the differences in drought tolerance between these two RIL seedlings under prolonged drought stress conditions.

### Differential expression profiles of aquaporins genes in drought-tolerant and -sensitive lines

The dynamic enrichment revealed that the GO “transmembrane transport” category underwent changes in RIL70 at 1 and 2 days after drought stress, and in RIL70 and RIL93 at 5 days after drought stress (Table S4). This category included 169 DEGs derived from RIL70 and RIL93 during drought stress. The *aquaporins* genes as a major functional group accounted for the most important part of this category. Among the 8 DEGs at 1 day after drought stress, two genes encoding *plasma membrane intrinsic proteins (PIPs)*, three genes annotated as *tonoplast intrinsic proteins* (*TIPs*) and two *NOD26-like protein* (*NIPs*) genes all showed decreased expression in RIL70, while none were differentially expressed in RIL93. Another gene encoding an unknown protein involved in transmembrane transport also showed decreased expression in RIL70 but was unchanged in RIL93. Among the 8 DEGs at 2 days after drought stress, the expression of three *PIP* genes, two *NIP* genes, a *TIP* gene and two putative *plasma membrane intrinsic protein* genes were all decreased in RIL70, but only one *PIP* gene decreased in transcript abundance in RIL93. At 5 days after drought stress, 22 DEGs encoding aquaporins showed expression changes in RIL70 and RIL 93. Among these genes, the expression of a *PIP* gene, two *NIP* genes, and two *putative plasma membrane intrinsic protein* genes was decreased, and a *TIP* gene (*GRMZM2G025122*) showed increased transcript abundance in RIL70. As shown in Table S4, four *PIP* genes, six *TIP* genes, and four *putative plasma membrane intrinsic protein* genes showed an increased expression profile and three *PIPs* and three *NIP* genes showed decreased transcript abundance in RIL93. Of the remainder of the 135 genes, only 20 genes showed expression changes in RIL70, and all of them showed mixed expression profiles in RIL93. Overall, these data suggest that different regulation of transmembrane transport genes may contribute to the differences in drought tolerance between these two RIL line seedlings under drought stress.

### Distant regulation of detoxification genes characterized the tolerance to drought stress

The dynamic enrichment showed that the GO “response to stress” was over-represented at 2 and 5 days after drought stress in the drought-sensitive line, but only at 5 days with a relatively lower overrepresented level after drought stress in the drought-tolerant line (Table S5). There were 21 DEGs derived from RIL93 distributed in this GO term at 2 days after drought stress; two late embryogenesis abundant domain-containing (LEA) protein-encoding genes and two heavy metal transport/detoxification superfamily protein-encoding genes showed increased transcript abundance. The other genes all showed expression changes in RIL93. Among 301 DEGs, the expression level increased in two dehydrins family proteins, heavy metal ATPase 5, five heavy metal transport/detoxification superfamily proteins, six LEA family proteins and four glycine-rich protein-encoding genes. Seven heavy metal transport/detoxification superfamily proteins, two heavy metal ATPases, two drought sensitive 1 proteins and a glycine-rich protein-encoding gene showed decreased transcript abundance in RIL93 at 5 days after drought stress. The other 134 and 137 genes involved in the process increased or decreased expression in RIL93. Compared to the DEGs in RIL93, the number of genes induced by drought stress was much less in RIL70. No gene changed at 2 days after drought stress, and there was a LEA family protein, a glycine-rich protein, a dehydrins family protein and a heavy metal transport/detoxification superfamily protein-encoding gene with increased expression levels, and two heavy metal transport/detoxification superfamily protein-encoding genes and a drought-induced gene with decreasing transcript abundance. The other 50 genes of this category showed mixed expression changes in RIL70 at 5 days after drought stress.

The genes annotated by the GO category “protein folding” (GO: 000645) were absent in the initial drought stress stage, and overrepresented at 2 and 5 days after drought stress in both lines (Table S6). In the 2 days after drought stress, there were 13 genes with an increased expression level, including five heat shock proteins, three ubiquitination-process related proteins, three chaperone protein-encoding genes, and two genes involved in protein degradation (*GRMZM2G115277* and *GRMZM2G109142*). Otherwise, five *heat shock protein* genes and a *chaperone superfamily protein* gene had a decreased expression level in RIL93. Only two genes showed lower transcript amounts, including a *heat shock protein* (*GRMZM2G024668*) and a *UBC protein* gene (*GRMZM2G156517*) in RIL70. These data suggested that this group of genes was more influenced by drought in RIL93 than RIL70. The difference between the RILs was more apparent at 5 days after drought stress. There were 72 genes with changed expression profiles; among them, nine heat shock proteins, four ubiquitination process-related proteins, nine chaperones and related proteins, four protein degradation-related-encoding genes and an ER protein-encoding gene showed increased transcript abundance, and another 6 ubiquitination process-related proteins, 8 chaperone proteins, 13 heat shock protein-encoding genes, and 16 protein folding member-encoding genes showed a decreased expression level in RIL93. There were only eight DEGs in RIL70, including a heat shock protein, a UBC protein and a chaperone protein-encoding gene with an increased expression level; the other four genes showed decreased transcript abundance. In this study, one of the most changed categories between the RILs was “oxidant reduction” (Table S7). There were 311 DEGs in this category in RIL70 and RIL93 under drought stress. Among them, none were present at the initial drought stress stage, but 40 and 271 DEGs were present at 2 and 5 days after drought stress, respectively. At 2 days after drought stress in RIL93, there were nine *peroxidase* genes and two *glutathione S-transferase* genes with increased expression levels, and two *peroxidase* genes and a glutathione *S-transferase*-encoding gene that were decreased. Only one *peroxidase* gene showed increasing transcript abundance in RIL70. For the other genes in the oxidation-reduction category, only seven genes showed expression changes in RIL70, while there were 19 genes with transcript changes in RIL93. The diverse trend of the expression profiles of the DEGs in this term between the two RILs became more obvious at 5 days after drought stress. Among 22 ROS scavenger genes, only three *peroxidase* genes, three *glutathione S-transferase* genes, and a *catalase* gene showed increased transcript abundance, and 15 genes encoding peroxidase, glutathione S-transferase, ascorbate peroxidase, and catalase showed decreased transcript abundance in RIL93. Except for a *peroxidase* gene, all had increased expression levels in RIL70.

These data suggest that the genes involved in detoxification signaling, such as response to stress, protein folding and oxidation-reduction, were more influenced by drought stress in the drought-sensitive line than the drought-tolerant line.

### Response to drought stress may be related to the regulation of the photosynthesis pathway

**Figure 6:**
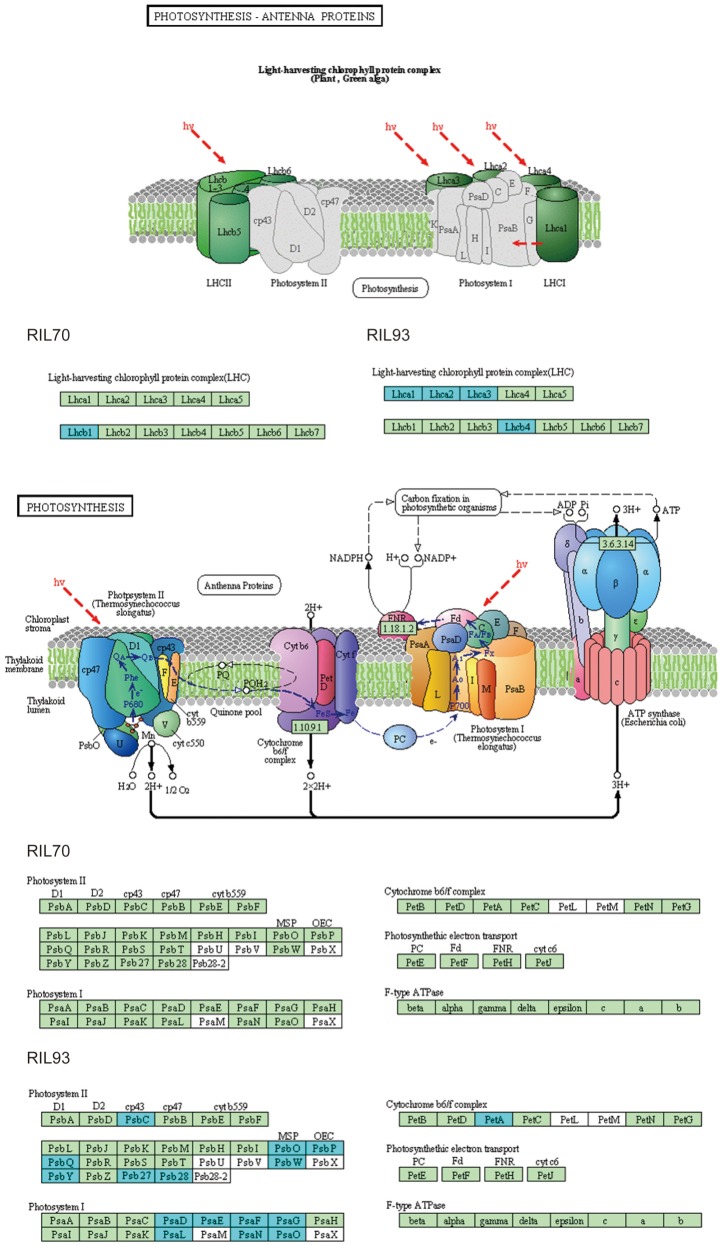
**KEGG map of the photosynthesis-antenna proteins and photosynthesis pathway between RIL70 and RIL93 in DT2**. This represents an analysis of DEGs, comparing drought-treated samples to untreated control. Boxes with a blue frame indicate that the corresponding DEGs were down-regulated in the drought-treated samples, and the boxes without any colored frame indicate that the expression levels of the corresponding genes were not changed, as determined by RNA-seq.

In this study, the different responses of the two extremely different RILs to drought stress were also observed in the biological process of photosynthesis (Figures 4, 6). Overall, this category presented obvious changes in RIL93 at 2 and 5 days after drought stress, and presented mild changes in RIL70 at 5 days after drought stress. The gene sets annotated by the category “photosynthesis” consisted of 32 DEGs in RIL93 at 2 days after drought stress (Table S8). Among these genes, five genes (*LHCA1, LHCB, LHCA3*, and 2 *LHCA2* genes) encoding subunits of the light-harvesting chlorophyll-protein (LHC) complex, which absorbs light and passes it to the light reaction center of the corresponding photosystem, showed decreased transcript abundance in RIL93. Only one LHC-encoding gene showed decreased expression in RIL70 at 2 days after drought stress. Another 13 genes (2 *PsbP, PsbQ2*, 2 *PsbQ2-like*, 2 *PsbX, PsbY, Psb28*, and 4 other genes) encoding the different protein subunits of the photosystem II reaction center pigment protein complexes (PS II-RC), which is the core of photosystem II and functions as the light reaction center, also showed decreased transcript abundance in RIL93. Otherwise, five genes (*PsaD2, PsaF, PsaG, PsaI*, and *PsaP*) encoding the different subunits of the photosystem I reaction center pigment protein complexes (PS I-RC), five genes involved in photosynthetic electron transport and the other four genes of this term also showed decreased transcript abundance in RIL93. However, these genes showed no significant changes in RIL70. The category “photosynthesis” consists of 65 DEGs in RIL93 at 5 days after drought stress (Figure S6, Table S8). These genes were assigned to different parts of the photosynthesis pathway, such as photosystem I, photosystem II, cytochrome (Cytb6f) complex and ATP synthase. A large group consists of 11 LHC-encoding genes (*LHCA1, 3 LHCA2, LHCA3, LHCA5, LHCBCP24, LHCB6*, and 3*LHCB4*), 16 PS II-RC-encoding genes (*PPL1, PSBO1, PSBO2, PSBP-1, PSBQ-2, PSBQ-like 3, PSBC, PSBR, PSBW, PSB27*, 3 *PSB28*, and 3 *PQL1*) and 12 PS I –RC-encoding genes (*PSAD-1*, 2 *PSAE2*, 2 *PSAF*, 2 *PSAG*, 2 *PSAL, PSAO*, and 2 *PSAN*) also showed decreased transcript abundance in RIL93. Furthermore, 15 members of photosynthetic electron transport chain-encoding genes (*FD3*, 4 *FD2*, 2 *FD-like, ferric reduction oxidase 7*, 2 *FNR, plastocyanin*, two *Chloroplast envelope membrane proteins*, and two *photosynthetic electron transfer A* genes), located down-stream of photosystem I functioning in the various steps of photosynthetic electron transport, and three genes encoding ATP synthase (*ATPB, ATPC*, and *ATPD*), which functions in the catalytic synthesis of ATP in the photosynthetic electron transport chain, all showed decreased transcript abundance in the RIL93 line. Otherwise, the other genes that belonged to this term showed a decreased expression level in RIL93 under drought stress. On the other hand, all of the genes listed above showed no significant changes, except for a *light harvesting complex mesophyll7* gene and two *ferredoxin-nitrite reductase* genes, in RIL70. The expression profiles of the genes involved in the photosynthesis pathway suggest that the system structure and the ability of photosynthesis is diminished in the drought-sensitive seedling line and is not affected in the drought-tolerant seedling line under the same stress conditions.

### Differential expression profiles of carbohydrate metabolism genes present in drought-tolerant and -sensitive lines

In this study, carbohydrate metabolism category genes showed altered expression patterns induced by drought stress in both of the maize RILs. As shown in Figure S7A, two genes encoding fructose-bisphosphate aldolase 2 (EC: 4.1.2.13) and two genes encoding transketolase (EC: 2.2.1.1) and NADP-malic enzyme 4 (EC: 1.1.1.40) showed decreased transcript abundance in RIL93, while no changes were observed in RIL70 at 2 days after drought stress. Among the DEGs at 5 days after drought stress (Figure S7B), two ribulose-bisphosphate carboxylase (rubisco, EC: 4.1.1.39)-encoding genes, two fructose-bisphosphate aldolase (EC: 4.1.2.13)-encoding genes, four transketolase (EC: 2.2.1.1)-encoding genes, a phosphoglycerate kinase (EC: 2.7.2.3)-encoding gene, a fructose-bisphosphate aldolase isozyme-encoding gene and a gene encoding NADP-malic enzyme 4 (EC: 1.1.1.40) were obviously repressed, while none of these genes changed expression level in RIL70. These results showed that the carbon fixation process might have been impaired due to the abolished photosynthesis system in RIL93 under severe drought stress. As shown in Table S9, the DEGs involved in starch and sucrose metabolism showed mixed changes in transcript abundance in RIL93. At 2 days after drought stress, two genes involved in starch synthase (*GRMZM2G046587* and *GRMZM2G121612*) showed decreased expression levels, a gene encoding β-amylase 3, which functions in starch degradation, showed increased transcript abundance and two genes encoding a sucrose transporter and a sucrose synthase, which take part in sucrose metabolism, showed increased transcript abundance in RIL93. A gene encoding cell wall invertase (*GRMZM2G139300*) also showed decreased transcript abundance in RIL93. The other genes included in this category showed decreased expression changes in RIL93, except for 5 genes. The genes listed above were not differently expressed in RIL70. Similarly, among the DEGs at 5 days after drought stress, two ADP glucose pyro-phosphorylase subunit-encoding genes and a starch synthase 2-encoding gene involved in starch synthesis showed decreased transcript abundance, while a phosphorylase domain containing protein-encoding gene, three beta-amylase 1-encoding genes and a dis-proportionating enzyme-encoding gene involved in starch degradation showed increased transcript abundance in RIL93. Two sucrose synthase-encoding genes and two sucrose transporter-encoding genes involved in sucrose metabolism also showed increased transcript abundance in RIL93. None of these genes, except for a starch degradation-related gene (*GRMZM2G099678*), showed significant changes in RIL70. Otherwise, two genes encoding cell wall invertase and a gene encoding HXK1 involved in the sucrose conversion reaction all showed decreased transcript abundance in RIL93. These data suggest that starch biosynthesis has been suppressed and starch degradation and sucrose accumulation have been enhanced in RIL93, with no comparable change in RIL70. The conversion of sucrose has also been suppressed, which may lead to a blockage of hexoses entering the glycolytic pathway in RIL93. The other 7 genes in this category showed increased expression changes in the two RILs at 5 days after drought stress.

### Cell cycle and programmed cell death genes display distinct expression profiles in the drought-tolerant and -sensitive lines

**Figure 7:**
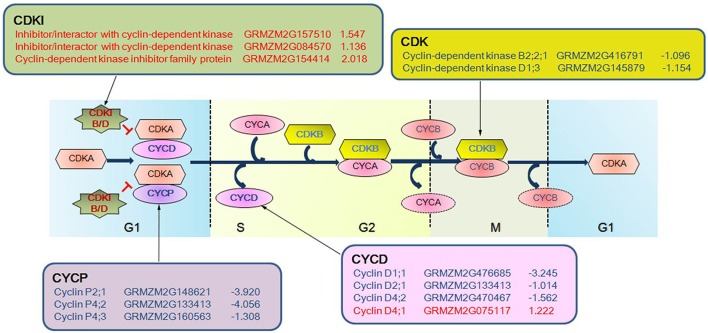
**The expression profiles of DEGs associated with cell cycle phases in drought-stressed RIL93**. Various symbols represent different groups of gene products. The oval indicates Cyclin and the hexagon and star indicate the Cyclin Dependent Kinase and Cyclin Dependent Kinase Inhibitor, respectively, where they exert an influence on the cell cycle. Blue and bold text in symbols indicates the corresponding DEGs that were down-regulated, and red and bold text indicates the corresponding DEGs that were up-regulated, as determined by RNA-seq in the drought-treated RIL93.

The cell cycle-related genes annotated by the homologous blast showed distinct expression patterns during drought stress between the two RIL maize seedlings (Figure 7). At 2 days after drought stress, two of the three *cyclins* showed decreased expression levels and *cyclin D5* (*GRMZM2G007130*) showed increased expression in RIL93, while no changes were present in RIL70. At 5 days after drought stress, all of the 10 *cyclins* except for *cyclin D4* (*GRMZM2G075117*) showed decreased transcript abundance in RIL93. The transcript levels of a *Cyclin/Brf1-like TBP-binding protein* gene and two genes annotated as *Cyclin-dependent kinase* were decreased in RIL93. Three genes encoding RAD-like 1, RAD9 and MRE11 all showed a decreased expression level, while another three genes with the same annotation as *cyclin-dependent kinase inhibitor* (*CDKI*) showed increased transcript abundance in RIL93. Only a *cyclin* gene (*GRMZM2G133413*) showed an increased expression level in RIL70 (Table S10).

The annotations of all transcript data were searched for programmed cell death gene terms across various species, including yeast, animals and plants. Of the identified genes, nine genes presented significant expression changes at 5 days after drought stress. Among these genes, two genes encoding metacaspase family proteins and an mlo family protein-encoding gene showed increased transcript abundance in RIL93. On the contrary, a lsd1 and the other five mlo family member-encoding genes showed decreased transcript abundance in RIL93. Consistent with the prediction, all of the DEGs mentioned above were not affected in RIL70.

### Quantitative real-time PCR (qRT-PCR) validation of cell wall biosynthesis, aquaporins related genes and DEGs randomly from RNA-Seq

**Figure 8:**
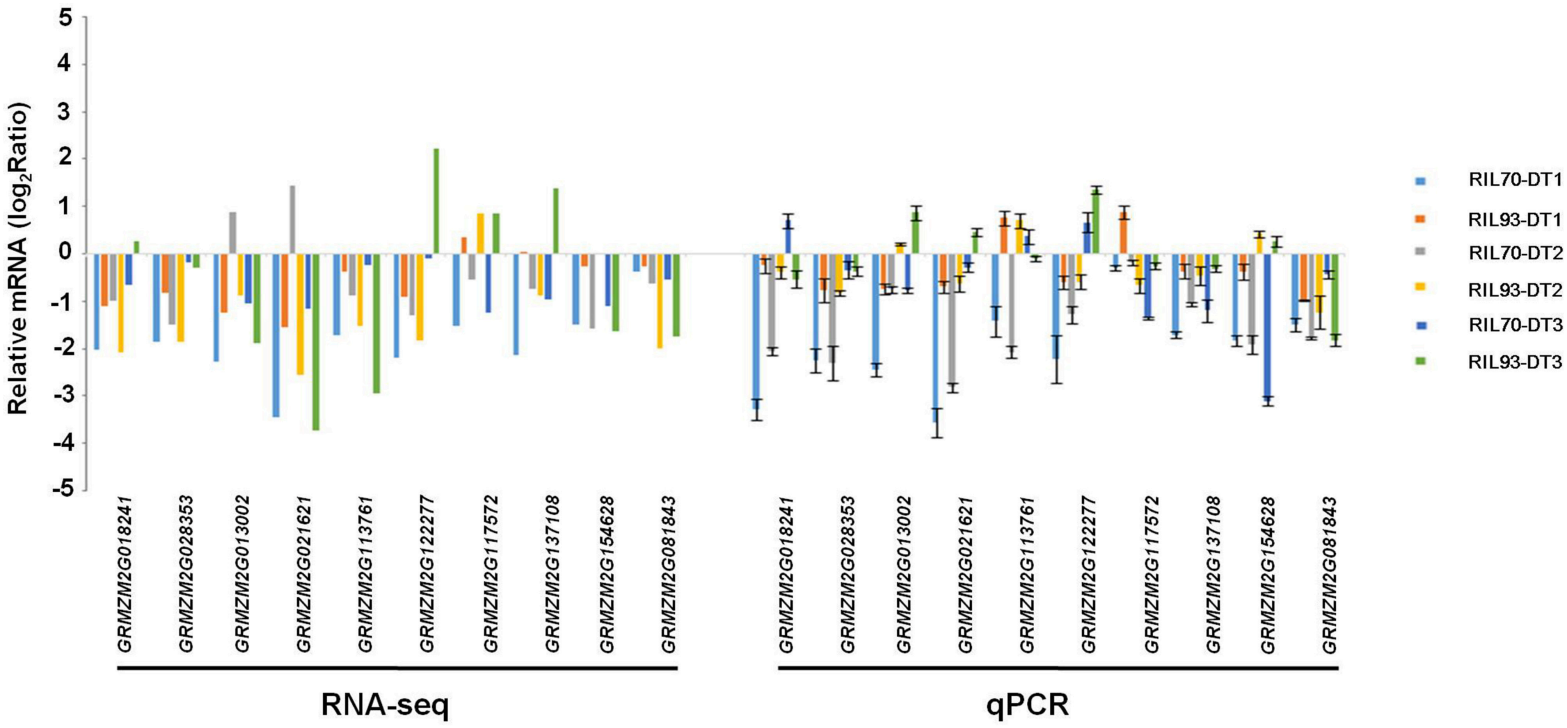
**Validation of RNA-seq results by RT-qPCR**. Quantitative RT-PCR validation of the expression patterns of maize cellulose synthase, expansion, and XTH encoding genes from different comparisons. The y-axis represents qPCR relative expression levels (log2fold-change) and fold-change of the RNA-seq data. Error bars represent the SE (*n* = 3).

To confirm the previous results and to confirm the accuracy and reproducibility of the RNA-Seq data, drought-stressed relative expression levels of six members of the cell wall biosynthesis genes, five genes encoding aquaporins and 16 DEGs that were randomly selected from RNA-seq results were detected by qRT-PCR in both RILs (Figure 8, Figure S8). The primers for these genes were designed using the Beacon Designer software (version 7.0).

## Discussion

### The distant drought response mechanisms underline RIL70 and RIL93

*Zea mays* is relatively sensitive to drought, and its growth is constrained due to drought stress. In this study, the strains RIL70 and RIL 93 presented distinct patterns of drought tolerance under drought stress (Figure 1, Figure S3). RIL70 had a significantly higher WRA than RIL93 (Figure S3), suggesting that the drought tolerance adaptation may include decreased water desorption. The results of CMS determinations were consistent with the WRA results, which suggest that the enhanced drought tolerance might be due to enhanced water retention capacity caused by turgor pressure augmentation. The maximum efficiency and actual efficiency of PSII photochemistry are measured as Fv/Fm and ΦPSII respectively, and together with the photosynthetic rate are a direct reflection of PSII activity and photosynthetic efficiency. Environmental stresses are associated with decreases in the Fv/Fm ratio and ΦPSII (Sui et al., 2015). In this study, RIL70 has more robust photosynthetic capabilities than RIL93 under drought, as suggested by lower Fv/Fm ratios and ΦPSII in RIL93 (Figure 2). Based on the phenotypic and physiological characteristics of the two RILs, we speculated that the enhanced drought tolerance in RIL70 is possibly due to an improvement in water retention caused by turgor adjustment and robust photosynthetic capabilities under drought stress.

### Differentially expressed genes of cell wall biosynthesis and transmembrane transport categories involved in turgor homeostasis determinate the distant tolerance to drought stress in both RILs

Plant cell walls consist of cellulose, a diverse group of different hemicelluloses such as xyloglucans that bind to cellulose and pectins, and the existing models suggest that the interaction of each xyloglucan polymer binding to at least two cellulose fibers is regulated by two groups of enzymes, expansions, and xyloglucan endotransglucosylases/hydrolases (XTH) (Cosgrove and Jarvis, 2012). The function of these two classical loosening enzymes, such as expansions or XTH, is similar to the action of cell wall loosening, which relieves the cross-linking of cell wall components leading to stiffening of the cellular structure accompanied by the cessation of the cell wall (Kieffer et al., 2000; Renew et al., 2005; Wakabayashi et al., 2012). Thus, relieving plant cells from water loss may occur by compensating for the changes in turgor pressure caused by the cessation of the cell wall during drought-induced osmotic stress (Bartlett et al., 2012; Krasensky and Jonak, 2012; Tenhaken, 2014). Several types of components functioning in cell wall metabolism pathways all showed decreased transcript abundance in RIL70 compared to RIL93 at 1 and 2 days after drought stress, indicating that the cell wall loosening and biosynthesis processes were crippled in drought-tolerant lines. These expression profiles will retard the expansion of the cell wall and protect the plant cells from water loss by relieving the decreased turgor caused by drought stress, which may lead to the maintenance of a stable state uninfluenced by drought stress over a long period in RIL70 compared to RIL93. Two previous studies revealed that genes involved in cell wall metabolism were generally repressed during osmotic stress in Arabidopsis (Bray, 2004; Wang et al., 2016) and that mutation of the cell wall biosynthesis genes showed a drought tolerant phenotype compared to the wild type in Arabidopsis (Chen et al., 2005). All of these facts indicated that the decreased expression profile of genes involved in cell wall biosynthesis resulted in better drought tolerance in RIL70 compared to RIL93.

Aquaporins are membrane channels that facilitate the transport of water across biological membranes of most living organisms. In plants, aquaporins promote water transport through cellular membranes, and play an important role in water homeostasis by turgor regulation (Maurel et al., 2015). Previous studies revealed that aquaporin-like proteins, also called major intrinsic proteins (MIPs), are divided into the PIP, TIP and NIP subfamilies, and the transcription of most MIPs in Arabidopsis was down-regulated in leaves upon drought stress (Alexandersson et al., 2005, 2010). It has been reported that various MIPs showed negative roles in the drought tolerant response, for the over-expression of these members was associated with a decreased resistance to salt or drought stress in different species, such as Arabidopsis, Barley and saltbush Atriplex canescens (Aharon et al., 2003; Katsuhara et al., 2003; Li et al., 2015). In our study, several types of *MIPs* all showed decreased transcript abundance in RIL70 at 1 and 2 days after drought stress, but only one *PIP* showed expression changes in RIL93 at 2 days after drought stress. This result suggested that a general down-regulation of AQPs might be a way to minimize water flow through cell membranes and uphold leaf turgor in drought-tolerant plants, and it might facilitate preventing the drought-tolerant line from being affected by drought stress with a stable state over a relatively long time compared to the drought-sensitive line, which further resulted in better drought tolerance of RIL70 compared to RIL93. Otherwise, the data indicate that the osmotic homeostasis induced by turgor changes results from the expression regulation of these two categories of genes during the initial stage of drought stress, and led to the distinct phenotypes between the two RILs under drought stress, which showed that RIL70 had a higher water relative content (WRC) and cell membrane stability (CMS) than RIL93 (Figure S3).

### The response of detoxification signaling to the drought stress were different in the two maize RIL seedlings

Drought stress affects multiple aspects of plant physiology and metabolism, and thus, numerous changes in gene expression can be predicted to occur. Some of the changes observed in this study are explained by osmotic stress signaling induced by changes in turgor, which led to the regulation of the genes involved in turgor homeostasis and water transport systems. Most of other changes induced by drought stress were identified as part of detoxification signaling caused by stress injury, protein denaturation and excessive accumulation of ROS, which initiates corresponding events such as the expression of drought response genes, e.g., LEA/dehydrins-type-encoding genes; changes in the expression level of genes encoding molecular chaperones and proteinases that remove denatured proteins; and the activation of genes encoding enzymes involved in the generation and removal of ROS and other detoxification proteins (Zhu, 2002). Dehydrins, a group of late embryogenesis abundant II proteins, play an important role in cellular responses to salinity and drought, preserving the stability of membrane-associated proteins and macromolecules, the adjustment of cytoplasmic osmotic pressure and the prevention of cell protein denaturation by the binding of water molecules to their surfaces (Heidari, 2008; Kosova et al., 2014). Similarly, glycine-rich protein, along with LEAs and dehydrins, plays a fundamental role in plant responses and adaptations to drought stress (Hanin et al., 2011). The expression of these hydrophilic, glycine-rich proteins is induced by dehydration in sensitive genotypes of other plant species, such as Bermuda grass and rice (Hanin et al., 2011; Zhao et al., 2011), and their expression also was associated with enhanced tolerance to drought stress in maize (Koag et al., 2003). The data described in the results indicated that a much more drastic drought injury response occurred in RIL93 than RIL70. Otherwise, many more genes involved in the response to stress process showed expression changes in RIL93 compared to RIL70. In addition, the results indicated that the genes involved in the repair system were more widely activated by stress injury in the drought-sensitive line compared to the drought-tolerant line.

Abiotic stresses such as drought and salinity usually cause protein dysfunction. Maintaining proteins in their functional conformations and preventing the aggregation of non-native proteins is particularly important for cell survival under stress. Molecular chaperones/heat shock proteins (Hsps) are key components, assisting in protein refolding under stress conditions and contributing to cellular homeostasis in cells under adverse growth conditions (Wang et al., 2004). Some Hsps/chaperones are induced by environmental stress conditions such as heat, cold, and drought (Lin et al., 2001; Sung et al., 2001), playing a crucial role in protecting plants against stress through the reestablishment of cellular homeostasis. The results (Figure 4, Table S6) indicated that protein dysfunctions caused by stress injury had occurred and the corresponding modification and degradation response was activated in RIL93, but was almost absent in RIL70 at the early stage of drought stress. Furthermore, this distinct response between the two RILs was more obvious at 5 days after drought. These data suggest that the process of protein folding was highly activated in RIL93 because of the protein denaturation caused by serious damage from drought in the drought-sensitive line compared to the drought-tolerant line.

In plants, the excessive accumulation of ROS can disturb cellular redox homeostasis, which leads to oxidative damage to cellular components and structures resulting from over-reduction of electron transport chain components in the mitochondria, plastids, and various detoxification reactions (Cruz de Carvalho, 2008). In our study, the expression data suggest that the antioxidant defense (oxidation-reduction) processes were present in RIL70, but were seriously abolished in RIL93 under the severe drought stress. Therefore, the ROS scavenging activity of antioxidant enzymes distributed in the chloroplasts and mitochondria was destroyed, in combination with the excessive accumulation of ROS caused by the disturbed photosynthesis and failed HXK activity. In this scenario, ROS damage is inevitable and will lead to the seedling death observed in RIL93. Drought stress did not impose a similar effect on RIL70 because of the unchanged and even slightly increased expression profiles of antioxidant enzyme-encoding genes. Overall, these data suggest that the genes involved in detoxification signaling such as response to stress, protein folding and oxidation-reduction were more influenced by drought stress in RIL93 compared to RIL70 as drought stress progressed, which specifically manifested as earlier initiated, higher intensity and more serious damage in RIL93 compared to RIL70. These changes in detoxification signaling genes may function as remedial measures to compensate for the failed turgor homeostasis caused by stress injury in RIL93 in the initial drought stage, which was absent or delayed in RIL70 during drought stress.

### The photosynthesis process presented diverse response in RIL70 and RIL93

Drought stress adversely impacts many aspects of the physiology of plants, especially photosynthetic capacity (Pinheiro and Chaves, 2011). Some genes in the photosynthesis pathway (Figure 6, Figure S6) showed decreased transcription abundance in RIL93, while, as expected, the opposite response was observed in RIL70. In the process of drought stress, DEGs encoding LCHA/B showed down-regulated expression in RIL93, which suggested that the absorption of light energy and the resulting photosynthetic electron transport process might have been limited in the drought-sensitive line under drought stress. Furthermore, the similar profile of DEGs encoding various components of the photosystem II protein complex and ATP synthase suggested that the binding stability of PS II was seriously affected and the electron transport mechanism was inhibited; thus, the photosynthetic electron transport process and the synthesis of ATP was blocked in RIL93 under drought stress. Overall, these transcription data indicated that the photosynthesis process was seriously affected due to the sharply decreased transcript abundance of a wide range of required genes, which resulted in a lower level of various fluorescence kinetics parameters and photosynthesis rate in RIL93 compared to RIL70 during drought stress (Figures 2, 3).

### Carbohydrate metabolism are more affected by drought stress in the RIL93

Carbohydrate metabolism occupies the core of bio-molecular metabolism, and the substrates involved in carbohydrate metabolism provide the essential saccharides and energy that the plant needs. The essential changes in the expression profiles of genes participating in carbohydrate metabolism may induce the adjustment and feedback that plants undergo during drought stress. Previous reports showed that the changed expression of genes involved in carbohydrate metabolism caused changes in the carbohydrate content in various plant species under drought stress (Arabzadeh and Khavari-Nejad, 2013). In our study, many types of enzymes in the multiple metabolic pathways involved in carbon movement and process assimilation were differentially expressed in the two extremely different lines under drought stress (Table S9). Among them, two main groups of genes involved in carbon fixation in photosynthetic organisms and starch and sucrose metabolism were very different between RIL70 and RIL93.

Ribulose-bisphosphate carboxylase (rubisco) is a key enzyme in carbon fixation. Rubisco catalyzes the incorporation of CO_2_ into ribulose 1,5-bisphosphate and plays an important role in CO_2_ assimilation. Two genes encoding Rubisco showed decreased transcripts in RIL93 but were not different in RIL70 at 5 days after drought stress, suggesting that the efficiency of CO_2_ assimilation had been influenced in the drought-sensitive line compared to the drought-tolerant line. These data were similar to previous studies in which CO_2_ assimilation was influenced by abiotic stress in other plants (Plaut and Federman, 1991). Otherwise, the expression of the two genes encoding NADP-ME decreased in RIL93 at 2 and 5 days after drought, but presented no changes in RIL70. NADP-ME is important for the carbon fixation pathway because it catalyzes the reversible oxidative decarboxylation of L-malate to produce CO_2_, pyruvate and NADPH (Rothermel and Nelson, 1989). The genes encoding NADP-ME were down-regulated in RIL93, which could reduce the content of CO_2_, pyruvate and NADPH, and further might reduce the efficiency of the dark reactions of photosynthesis. Thus, carbohydrate fixation was blocked in the drought-sensitive line under drought stress.

In addition to the changes in the genes above, other genes encoding starch biosynthesis enzymes showed declines in expression level, while those encoding starch degradation enzymes increased in RIL93 at 2 and 5 days after drought stress. This result suggested that carbohydrate assimilation might be affected by the breakdown of photosynthesis under stress. Many types of sugars were provided for the requirement of the drought stress response in the drought-sensitive line. All these changes were nearly absent in the drought-tolerant line. The changes in the expression profiles of the three Suc synthase-encoding genes agrees with this conclusion, and indicates the possibility of Suc hydrolysis and the use of hexose as the primary sugar in the drought-sensitive line under drought stress.

The cell wall invertases (CWINs) are one component of plant invertase (INV) enzymes, which hydrolyzes sucrose into glucose and fructose, thereby playing key roles in primary metabolism and plant development. Previous studies revealed that CWIN affects the process of cell division in embryos through glucose-mediated signaling, leading to reduced cell number and cell size in fruit from various species (Ott, 1976; Vilhar et al., 2002). An equivalent result was observed in our data, where the expression of Incw2 and the isoform of Incw2 was decreased (Table S8), the cell cycle appears to be blocked (Figure 7), the release of hexose to the nutrient supply for the drought stress response requirement was inhibited, and ultimately the drought sensitive phenotype appeared in RIL93 (Figure 1). Hexokinase (HXK) plays an important role in Glc metabolism, which modulates many physiological processes in plants (Rolland et al., 2006). Family members such as HXK1 function as a key enzyme that continuously receives a supply of Glc, catalyzing the conversion of ATP to ADP, and allowing the normal operation of flux in the mitochondrial electron transport chain and avoiding excessive ROS accumulation by maintaining its activity at a constant level (McFarlin and Koprowski, 1990). Our data show that the expression of the maize homolog of HKX1 is decreased in RIL93, which indicates that hexokinase could not function metabolically because of its reduced activity caused by the decreased level. In turn, this may cause the accumulation of ROS in the mitochondrion and other subcellular locations; this, along with the loss of the ROS scavenging system, leads to ROS damage because of that excessive accumulation of ROS in RIL93. Evidence of similar events was not present in RIL70 under drought stress. Under these conditions, the ROS damage and failure of the supply of hexose for the stress response requirement led to the earlier appearance of the stress susceptibility phonotype in RIL93 compared to RIL70 under prolonged stress.

### Cell cycle and PCD processes responsed differentially in RIL70 and RIL93

Distinct responses of the two RILs to drought stress were also observed in the cell cycle process. A group of genes encoding cell cycle components promoting cell cycle progression presented decreased expression profiles, and the factor that inhibits cell cycle progression showed an increased transcript abundance in RIL93 (Figure 6). Cyclins are regulatory proteins that interact with cyclin-dependent kinases (CDKs) to control progression through the cell cycle. B-type cyclins are mainly present at the G2-M transition and M phase, and D-type cyclins play a role in the regulation of the G1-S transition (De Veylder et al., 2007). A novel class of seven cyclins designated as P-type cyclins (CYCPs) are binding partners of CDKA that participate in both cell division and the response of cells to nutrients in proliferating cells, and in differentiating and mature tissues (Torres Acosta et al., 2004). Various combinations composed of different cyclins and CDKs ensure normal cell cycle progression in different stages. In our data, a cyclin P- and a cyclin D-encoding gene showed decreased expression in RIL93, but presented no changes in RIL70 at 2 days after drought stress (Table S10). All of the *cyclin* genes except *cyclin D4* showed decreased transcript abundance in RIL93 at 5 days after drought stress, and only a *cyclin D2* had enhanced transcript abundance in RIL70. *CDKB*, which encodes a cyclin-dependent protein kinase, binds to cyclin CYCA and CYCB, involved in the regulation of the late S to M transition of the mitotic cell cycle (Inze and De Veylder, 2006; Van Leene et al., 2010). This family had a relatively decreased expression in RIL93 compared to RIL70 at 2 and 5 days after drought stress. CDKIs are important negative regulators of cyclin-dependent kinases (CDKs), which positively control the cell cycle during plant development. Previous studies showed that they controlled the transition from the mitotic cell cycle to the endo-reduplication cell cycle and promoted G1 arrest (Sabelli and Larkins, 2009). *KRP3*, a member of the CDKIs family in Arabidopsis, is an inhibitor of the 26S proteasome and has negative regulatory roles in the cell cycle and endo-reduplication in the SAM and leaves (Jun et al., 2013). Three homologs of *KRP3* in maize (*GRMZM2G157510, GRMZM2G084570*, and *GRMZM2G154414*) showed increased expression levels in RIL93, which indicated a disturbed cell cycle progression in RIL93 at 5 days after drought stress. On the other hand, a *CDKI* member presented decreased transcript abundance in RIL70. Furthermore, other genes, such as *RAD* and *MRE* family members, which are negative factors in cell division during plant growth and development (Heitzeberg et al., 2004; Samanic et al., 2013), all declined in RIL93. This result indicated that a deficiency in meiosis might occur and led to the defective growth in RIL93 in the next prolonged drought.

The fluctuating changes in expression profiles of a group of cell cycle-related genes indicated an obvious perturbation to the cell cycle, which will devastate cell proliferation capabilities and lead to the seedling death phenotype in RIL93. Moreover, it is reported that sugars activate key cell cycle regulators and influence the regulation of the G2-M transition in plants (Skylar et al., 2011). The expression of sugar metabolism genes indicated that disrupted sugar metabolism under drought stress might additionally block the cell cycle in the drought-sensitive line.

Programmed cell death is an active, genetically controlled process in which damaged cells are selectively eliminated in a highly orchestrated, multi-step pathway through the involvement of specific proteases and nucleases (Petrov et al., 2015). In general, lower doses of ROS function as a signal in stress response, and higher concentrations may pose a significant threat that may eventually lead to programmed cell death (Gechev and Hille, 2005). It is reported that prolonged drought stress is often concomitant with ROS accumulation, mainly due to the increased electron leakage to triplet oxygen which may eventually lead to PCD (Gechev et al., 2012). In our data, a few homologs of several known PCD genes were identified that showed significant expression changes under drought stress (Table S11). *Metacaspases*, which are cysteine-dependent proteases, are required as a positive factor for oxidative stress-induced PCD, and the oxidative stress-induced PCD exhibited an important example of a metacaspase-dependent process that is conserved in evolution from protozoa to plants (Tsiatsiani et al., 2011). Two homologs of *metacaspases* showed an increased expression level in RIL93, but presented no change in RIL70. Lsd1 (lesions simulating disease resistance response) function as a negative regulator of cell death in the hypersensitive response and is suspected to reduce cell death spread (Dietrich et al., 1994). Its homolog (*GRMZM2G055135*) had an obvious decreased expression level in the RIL93, and as above, no changes occurred in RIL70 under drought stress. The mlo (modulator of defense and death) family members are broadly involved in cell death protection and in responses to biotic and abiotic stresses. Mutant studies in barley demonstrated that these genes were negative regulators of PCD (Piffanelli et al., 2002). Five of the six *mlo* homologous genes that met the cutoff showed a decline in expression level in RIL93, but presented no changes in RIL70 at 5 days after drought stress. In conclusion, the expression profiles of these PCD-related genes indicated that the progression of PCD was enhanced in RIL93 under drought stress, which might accelerate the survival of the drought-sensitive line during prolonged drought stress.

### Differentially expressed genes implicated in ABA response

ABA, a plant hormone, occupies core status in the drought response signaling network (Cutler et al., 2010). Previous studies showed that the level of ABA was increased in maize in response to drought stress (Jiang et al., 2011). In our study, the expression data for ABA synthesis genes indicated that the ABA biosynthesis process was enhanced (Table S12), and the induction in ABA may occur in RIL93. It also has been reported that severe drought stress can increase the production of ABA and subsequently up-regulate the expression of ABA responsive genes in plants (Mehrotra et al., 2014). As in our data, the expression of most bZIP genes is increased in RIL93, and nearly all of them presented a relatively higher expression level compared to RIL70. The increased expression of the genes involved in ABA synthesis and the ABRE transcription factors combined with other ABA responsive related proteins suggests that the ABA-mediated response pathway was completely activated and the expression of many genes that play an important role in drought responses was broadly induced under the conditions of drought stress in RIL93. However, most of these genes presented no changes and this effect was not observed in RIL70, which might indicate a stronger drought tolerance ability and a relatively higher threshold of drought tolerance in the drought-tolerant line compared to the drought-sensitive line.

## Conclusions

**Figure 9:**
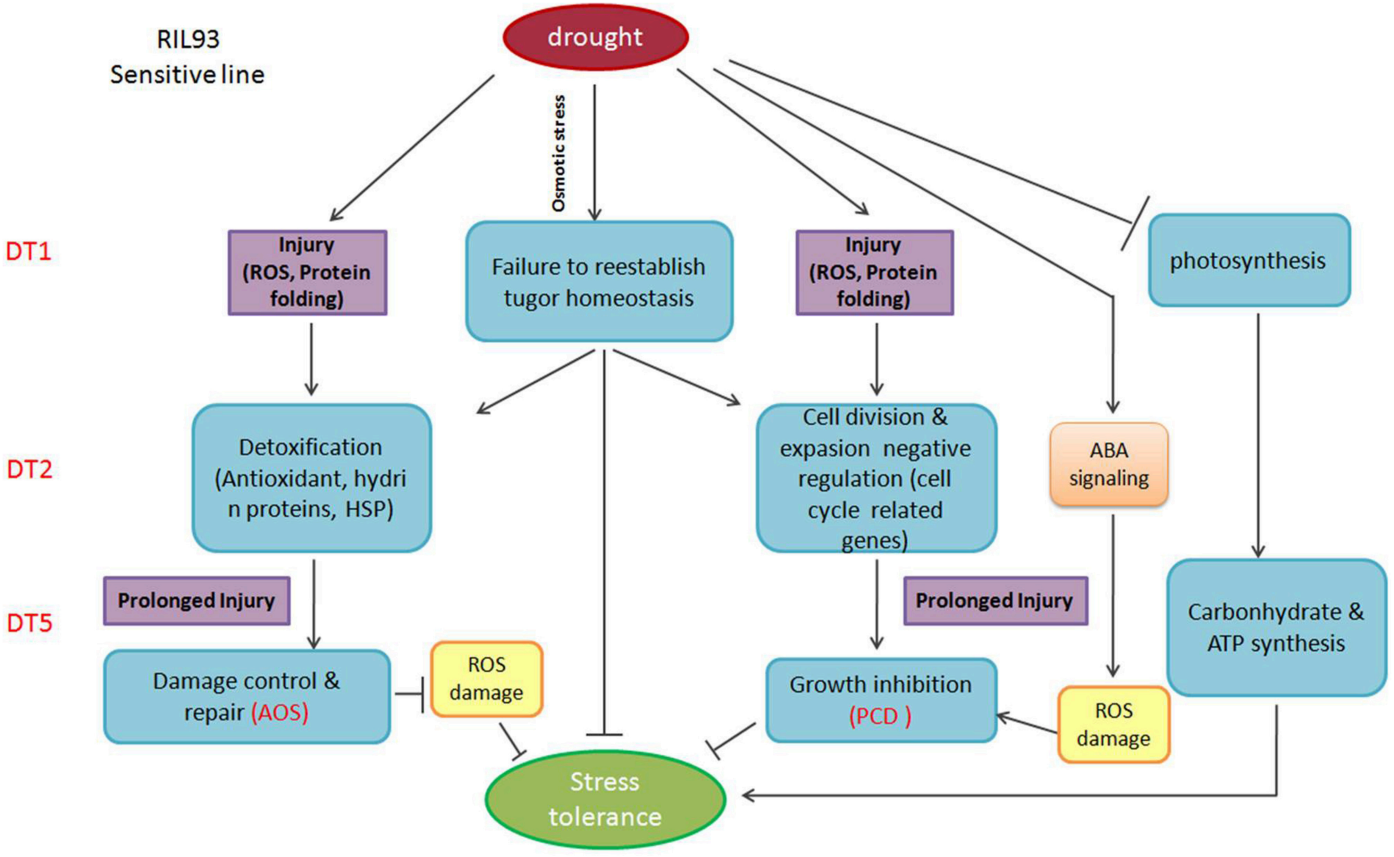
**Overview of the signaling pathways in RIL93 (drought-sensitive line), during the dynamic process of drought stress**.

The overall understanding of the drought tolerant mechanism in maize seedlings is limited for a long time. With the development of RNA-seq technology, it is expected to be a powerful method to reveal this mechanism. Through the analysis of the transcript level by RNA-seq in the two RILs with extremely different drought tolerance, our study identified that the osmotic homeostasis regulated by turgor changes caused by the expression regulation of cell wall biosynthesis and aquaporin-related genes, functioned as the first defense against drought and played an important role in the whole process of drought stress (Figure 9, Figure S9). The regulation of this homeostasis affected a seriers events such as the working of ROS-scavenging system, the operation of photosynthesis and the cell cycle process and the repression of PCD by regulating the subsequent downstream detoxification signaling members, then it caused the corresponding drought responsive phenotypes. Some potentially turgor changes related genes were highlighted to reveal their importance in regulation of drought tolerance. Otherwise, this study provided a foundation for the further understanding of the maize drought tolerant mechanism, and provided the valuable turgor changes related genes for promoting the development of drought tolerant genetic improvement breeding.

## Materials and methods

### Plant material and drought stress conditions

Seeds of two maize RILs, RIL70, and RIL93, which were derived from the F1 cross of the Ph4CV and F9721 parental lines, were used as the experimental materials in this study. RIL70 is considered to be tolerant to drought stress, while RIL93 is sensitive to salt stress (Figure 1). After being soaked with water at 28°C for 36 h, plump seeds were selected and sown in two types of pots (5.5 × 5.5 × 5 cm, four seeds in each pot; 10 × 10 × 5 cm, 10 seeds in each pot) filled with a 1:1 mix of vermiculite: nutrient soil and irrigated with tap water. After germination, the seedlings were cultured in controlled greenhouse conditions of 28/22°C (day/night) at a light intensity of 600 μmol m^−2^ s^−1^ (16-h-light/8-h-night) and 40–50% relative humidity and were irrigated daily to maintain soil water content close to field capacity for approximately 12 days until drought treatment. The three-leaf seedlings of the two RILs were treated with natural drought stress without water, until indications of withering were visible in the drought-sensitive seedlings, when the two RILs seedlings were re-watered. During this stage, from the start to 5 days after drought stress, the leaves tissues of the two RILs seedlings were harvested and samples labeled CK, DT1, DT2, and DT5 were used for RNA extraction and Illumina deep sequencing. In addition, the phenotypes and physiological indexes of the seedlings were detected.

### Measurement of RWC and CMS

For relative water content (WRA) measurements, 1 g of fresh leaves was cut into 1 cm fragments and weighed for the determination of fresh weight (FW). The proportions of fresh weight loss were calculated relative to the initial plant weights. The plants were finally oven-dried to a constant dry weight (DW) for 24 h at 80°C. WRA was measured according to the formula: WRA (%) = (FW − DW)/FW × 100.

Plant cell membrane stability (CMS) was determined with a conductivity meter (DDS-1, YSI), CMS (%) = (1-initial electrical conductivity/electrical conductivity after boiling) × 100. An equal weight (0.5 g) of leaves from the same position were removed from the seedlings before stress and treated from 1 to 5 days after drought stress. After splicing into 0.5-cm fragments, the tissues were immediately thoroughly rinsed with double distilled water (ddH_2_O) prior to immersion in 20 mL ddH_2_O at room temperature. After 12 h, the initial conductivities of the solutions were recorded. The samples were then boiled for 30 min, cooled to room temperature, and the final conductivities were measured.

### Measurement of chlorophyll fluorescence and photosynthesis

The chlorophyll fluorescence and photosynthesis were measured from 0 to 5 days after the seedlings underwent drought stress. For each treatment, the parameters of Chl fluorescence were measured independently on 12 plants. Measurements were taken on the second mature leaves of each of the chosen plants. Chl fluorescence was measured using a portable photosynthesis system (LI-COR LI-6400 XTR) following the protocol as described. Leaves had been pre-darkened for at least 1 h to determine the minimal and the maximal fluorescence. Minimal fluorescence (Fo) with all PSII reaction centers open was determined by modulated light that was low enough not to induce any significant variable fluorescence (Fv). Maximal fluorescence (Fm) with all reaction centers closed was determined by 0.8 s saturating light of 8000 μmol m^−2^ s^−1^ on a dark-adapted leaf. Then, the leaf was illuminated by an actinic light of 500 μmol m^−2^ s^−1^. Steady-state fluorescence (Fs) was recorded when the leaf reached steady-state photosynthesis. A second 0.8 s saturating light of 8000 μmol m^−2^ s^−1^ was given to determine maximal fluorescence in the light-adapted state (Fm'). Maximal photochemical efficiency (Fv/Fm) of PSII was expressed as: Fv/Fm = (Fm–Fo)/Fm. The quantum yield of PSII electron transport was: ΦPSII = (Fm'–Fs)/Fm'. Photosynthetic rates were also measured using a Li-6400 photosynthesis measurement system under the conditions as follows: photosynthetic photon flux density, 1000 μmol m^−2^ s^−1^; and chamber CO_2_ concentration, 400 μmol CO_2_ mol^−1^. Measurements were taken on the mature leaves of each of the chosen plants.

### Total RNA extraction, library construction and illumina sequencing

Total RNA was isolated from the leaves of two lines in different periods using a Total Plant RNA Extraction Kit (Karroten, Beijing, China) following the manufacturer's protocols. RNA degradation and contamination was monitored on 1% agarose gels. RNA purity was checked using the NanoPhotometer® spectrophotometer (IMPLEN, CA, USA). RNA concentration was measured using the Qubit® RNA Assay Kit in a Qubit® 2.0 Flurometer (Life Technologies, CA, USA). RNA integrity was assessed using the RNA Nano 6000 Assay Kit of the Bioanalyzer 2100 system (Agilent Technologies, CA, USA). A total amount of 3 μg RNA per sample was used as input material for the RNA sample preparations. Sequencing libraries were generated using the NEBNext® Ultra™ RNA Library Prep Kit for Illumina® (NEB, USA) following the manufacturer's recommendations, and index codes were added to attribute sequences to each sample. The library was sequenced on an Illumina Hiseq 2000 platform and 100 bp paired-end reads were generated.

### Differentially expressed genes (DEGs) detection and function analysis of DEGs

Reference genome B73 and genes annotation files were downloaded from the Phytozome website. RNA-seq clean reads were aligned to the reference genome using TopHat v2.0.9 (Trapnell et al., 2009) allowing a 2 segment mismatch. HTSeq v0.6.1 (Anders et al., 2015) was used to count the reads numbers mapped to each gene. Then, the Reads Per-Kilobase of the exon model per Million mapped reads (RPKM) of each gene was calculated based on the length of the gene and reads count mapped to this gene. Differential expression analysis for each sample was performed using the DESeq R package (1.10.1) (Anders and Huber, 2010) Genes with fold changes ≥2 and a false discovery rate (FDR) adjusted *P* < 0.05 were assigned as differentially expressed.

GO term enrichment analysis of DEGs was performed by AgriGO web server analysis tools (http://bioinfo.cau.edu.cn/agriGO). GO terms with *P*-value less than 0.05 and FDR (adjusted *P*-value) less than 0.1 were considered significantly enriched in our study (Du et al., 2010). The significantly enriched GO terms were used to draw a heat map by R script. The significantly enriched GO terms were used to draw a heat map by PERL script. KEGG pathways analysis of DEGs was performed using the KOBAS 2.0 web server (http://kobas.cbi.pku.edu.cn/) (Xie et al., 2011) and the KEGG web server (http://www.kegg.jp/) (Kanehisa and Goto, 2000).

### Quantitative real-time PCR analysis

Real-time PCR was performed in an optical 96-well plate with a Bio-Rad iQ5 thermo cycler (Bio-RAD, California, USA). Sixteen DEGs were randomly selected for quantitative real-time PCR to verify the RNA-seq results. Another 11 genes encoding cell wall biosynthesis and aquaporin-related proteins were also confirmed by qPCR. Primers for these genes were designed using the Beacon Designer software (version 7.0) (Table S13). One μg of total RNA was used per 20 μl reaction for reverse transcription. Each reaction mixture contained 10 μl SYBR Premix Ex Taq (Bio-RAD, California, USA), 0.4 μl of each primer, and 1 μl of 1:10 diluted template cDNA in a final volume of 20 μl. The PCR reactions were performed under the following thermal cycling conditions: 95°C for 30 s and 40 cycles of 95°C for 15 s, 56°C for 15 s, and 72°C for 30 s. The comparative C_T_ method of quantitation (Livak and Schmittgen, 2001) was used with maize β-tubulin as an internal standard. Three independent biological replicates of each sample and three technical replicates of each biological replicate were used for real-time PCR analysis. The relative fold change for each of the selected genes was determined for both the control and the drought-stressed seedlings.

## Author contributions

HM and QX initiated and designed the research; HM, SW, MS, and XS prepared the RNA-seq material; HM and CC performed all the experimental and bioinformatics analysis while HM and HC created maize ril lines. HM and QX wrote this manuscript. All of the authors discussed the results and commented on the manuscript. QX approved the final version of the article to be published.

### Conflict of interest statement

The authors declare that the research was conducted in the absence of any commercial or financial relationships that could be construed as a potential conflict of interest.
