# Supplementary figures and images for: Identification of Drought Tolerant Mechanisms in Maize Seedlings Based on Transcriptome Analysis of Recombination Inbred Lines
Source: Front Plant Sci. 2016 Jul 26;7:1080. doi: 10.3389/fpls.2016.01080 (PMC4961006; doi:10.3389/fpls.2016.01080)

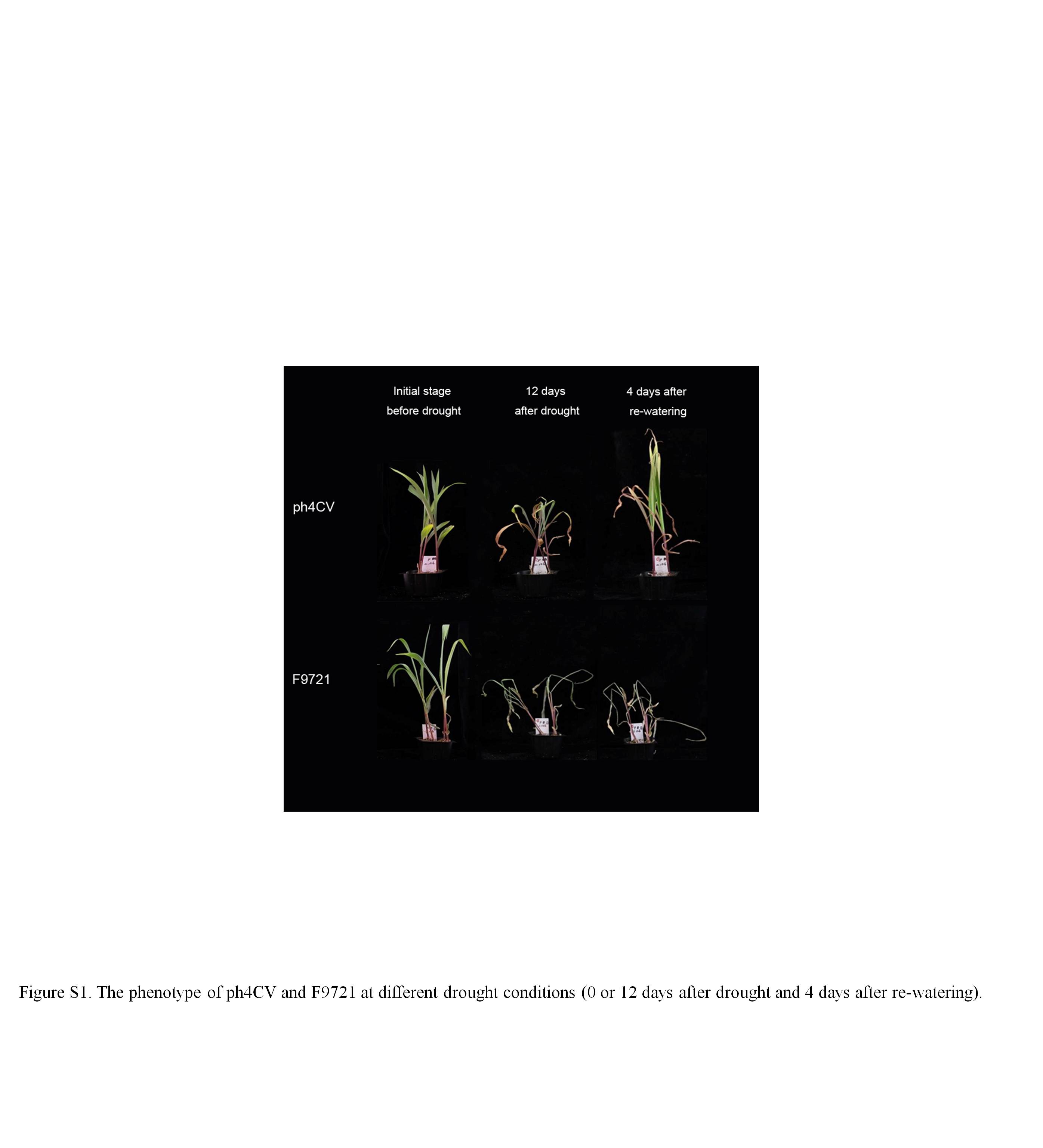

Supplement: Supplementary file 1 [file Image1.JPEG]

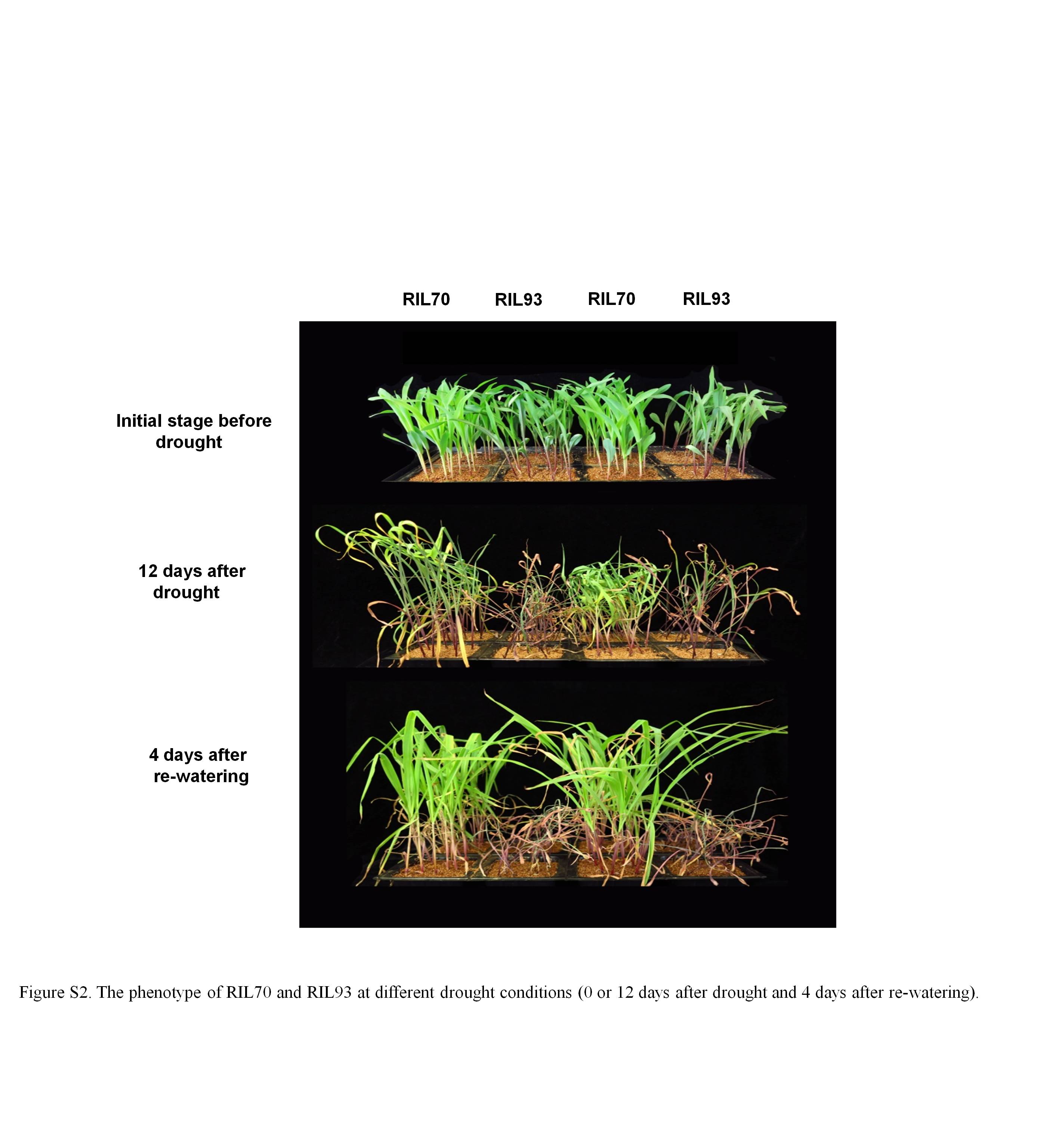

Supplement: Supplementary file 2 [file Image2.JPEG]

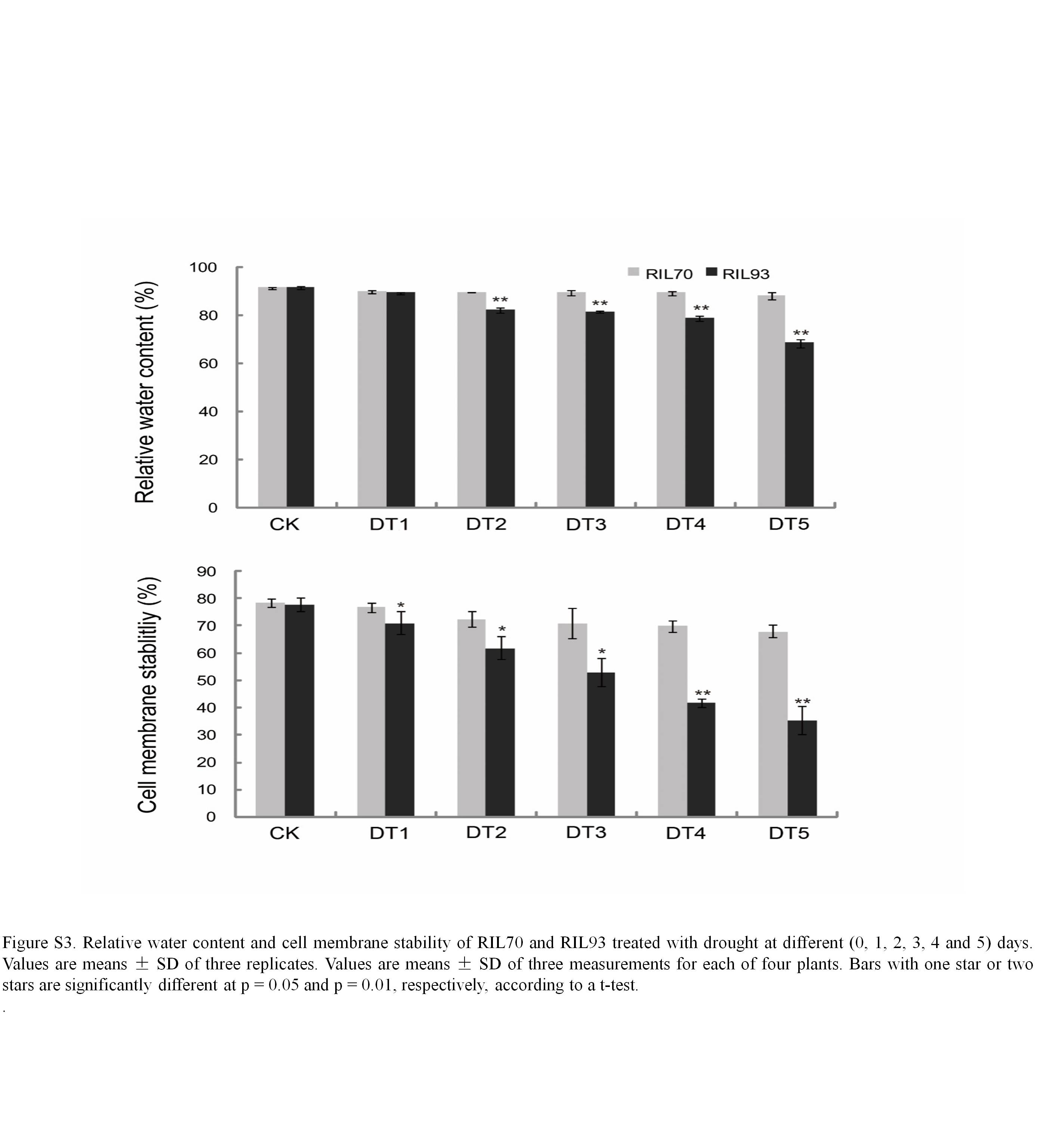

Supplement: Supplementary file 3 [file Image3.JPEG]

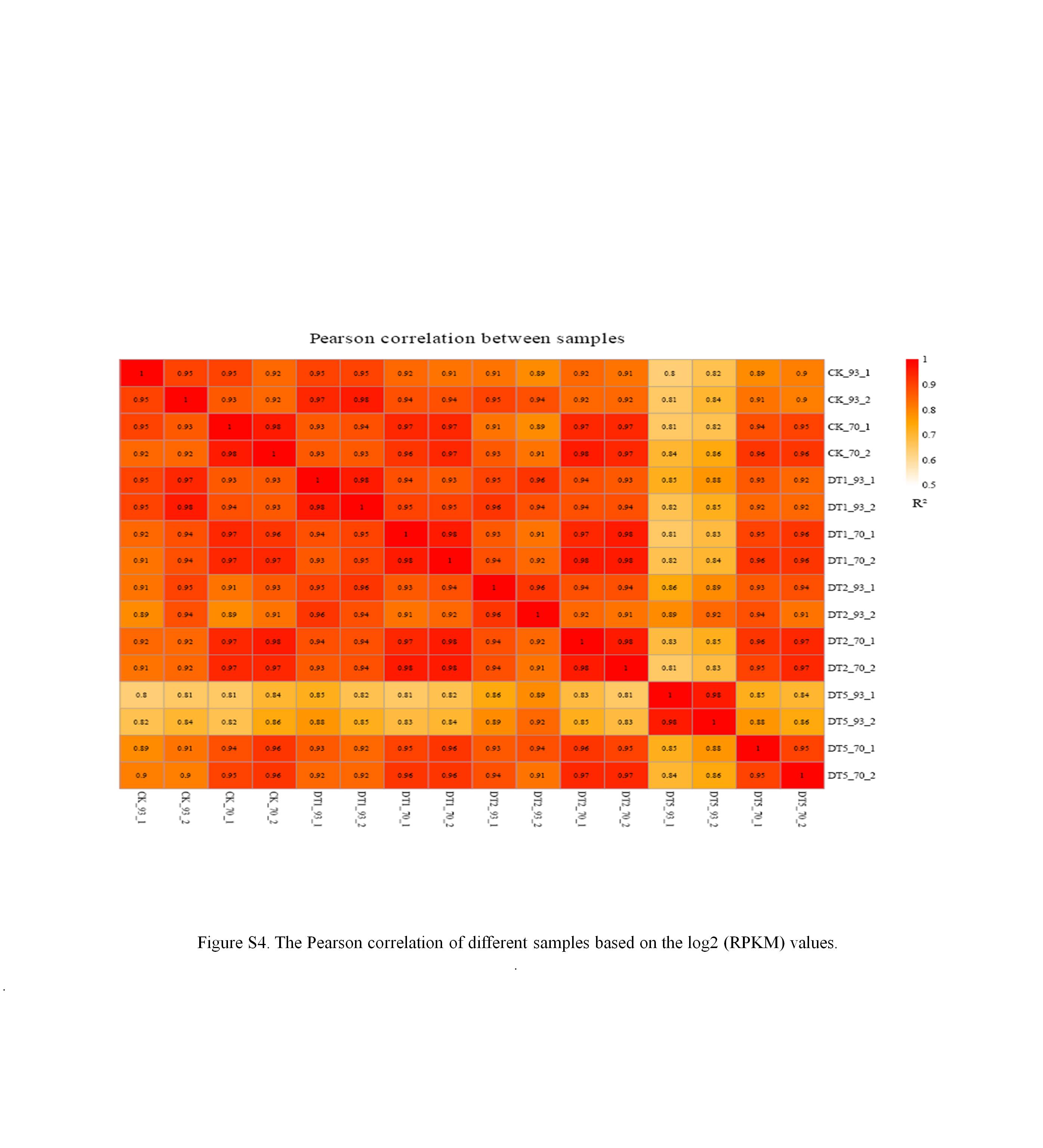

Supplement: Supplementary file 4 [file Image4.JPEG]

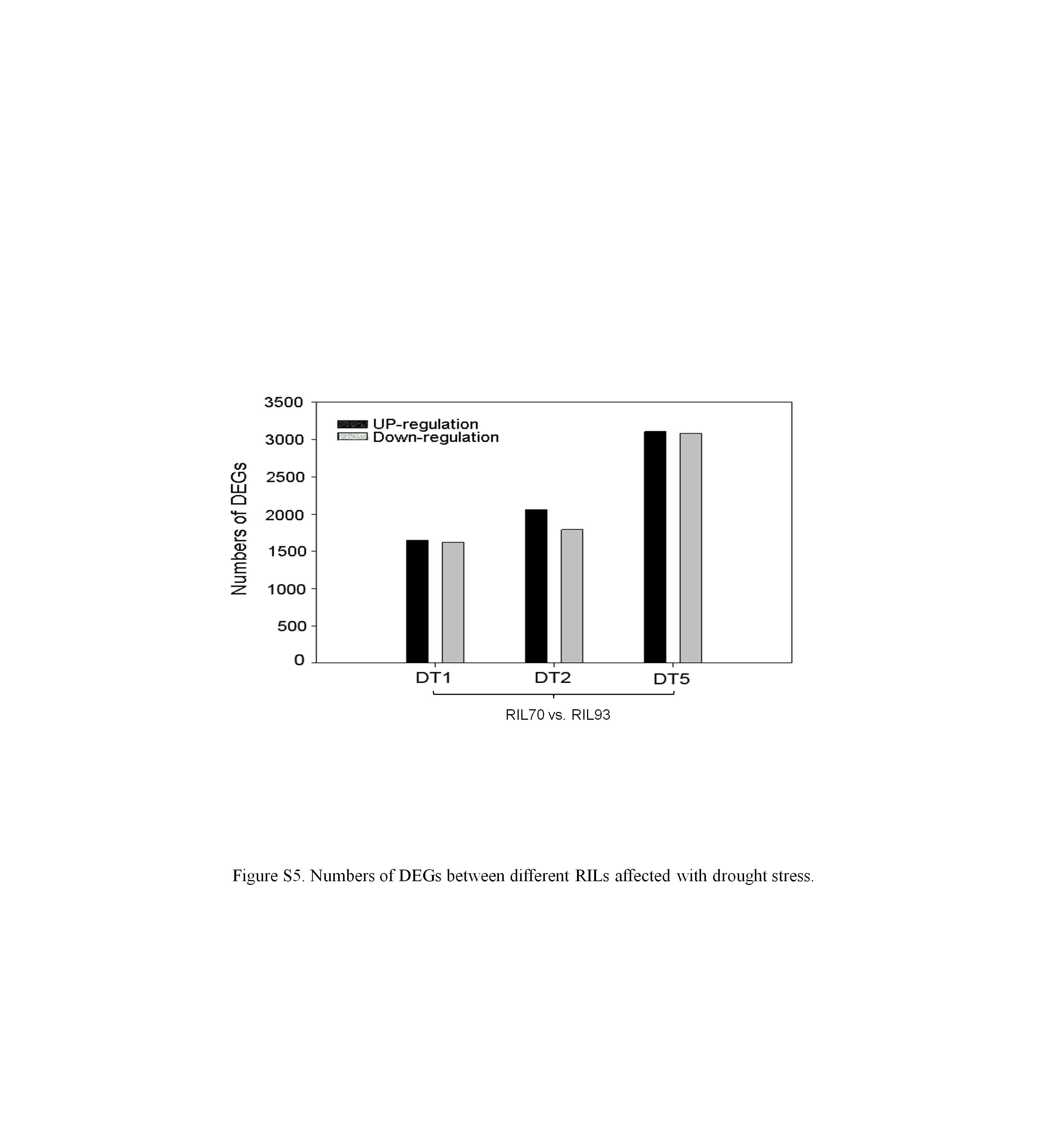

Supplement: Supplementary file 5 [file Image5.JPEG]

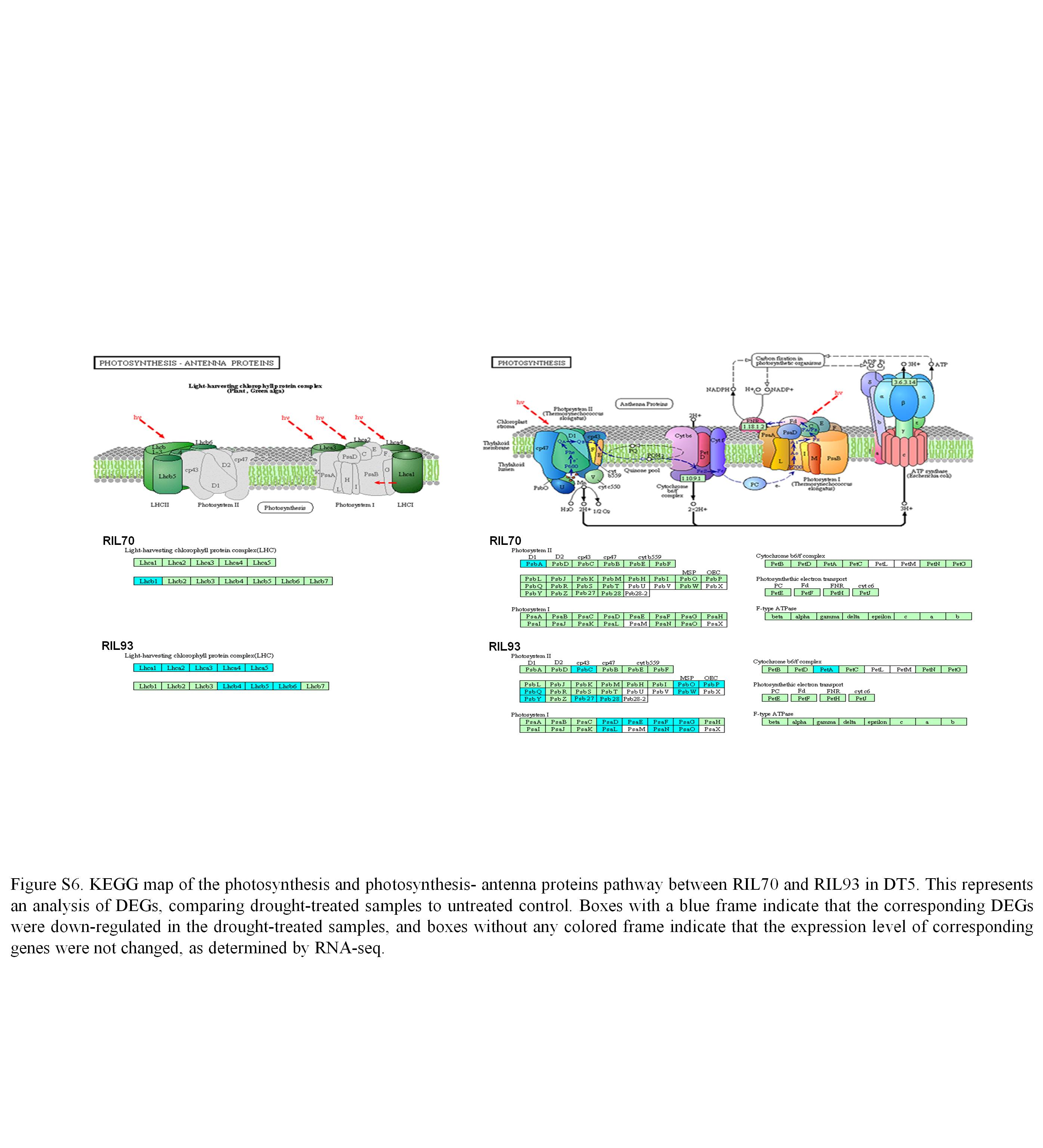

Supplement: Supplementary file 6 [file Image6.JPEG]

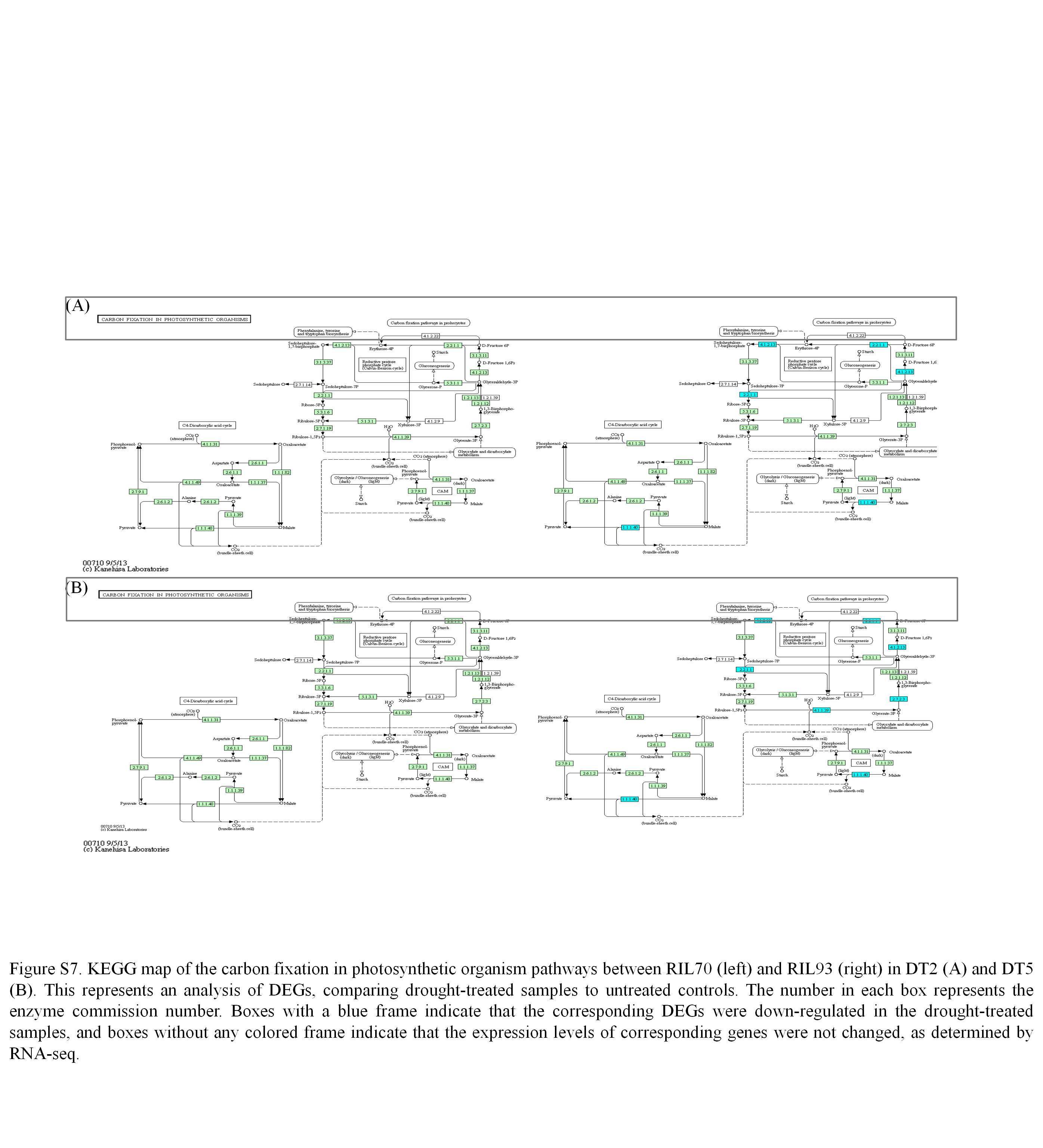

Supplement: Supplementary file 7 [file Image7.JPEG]

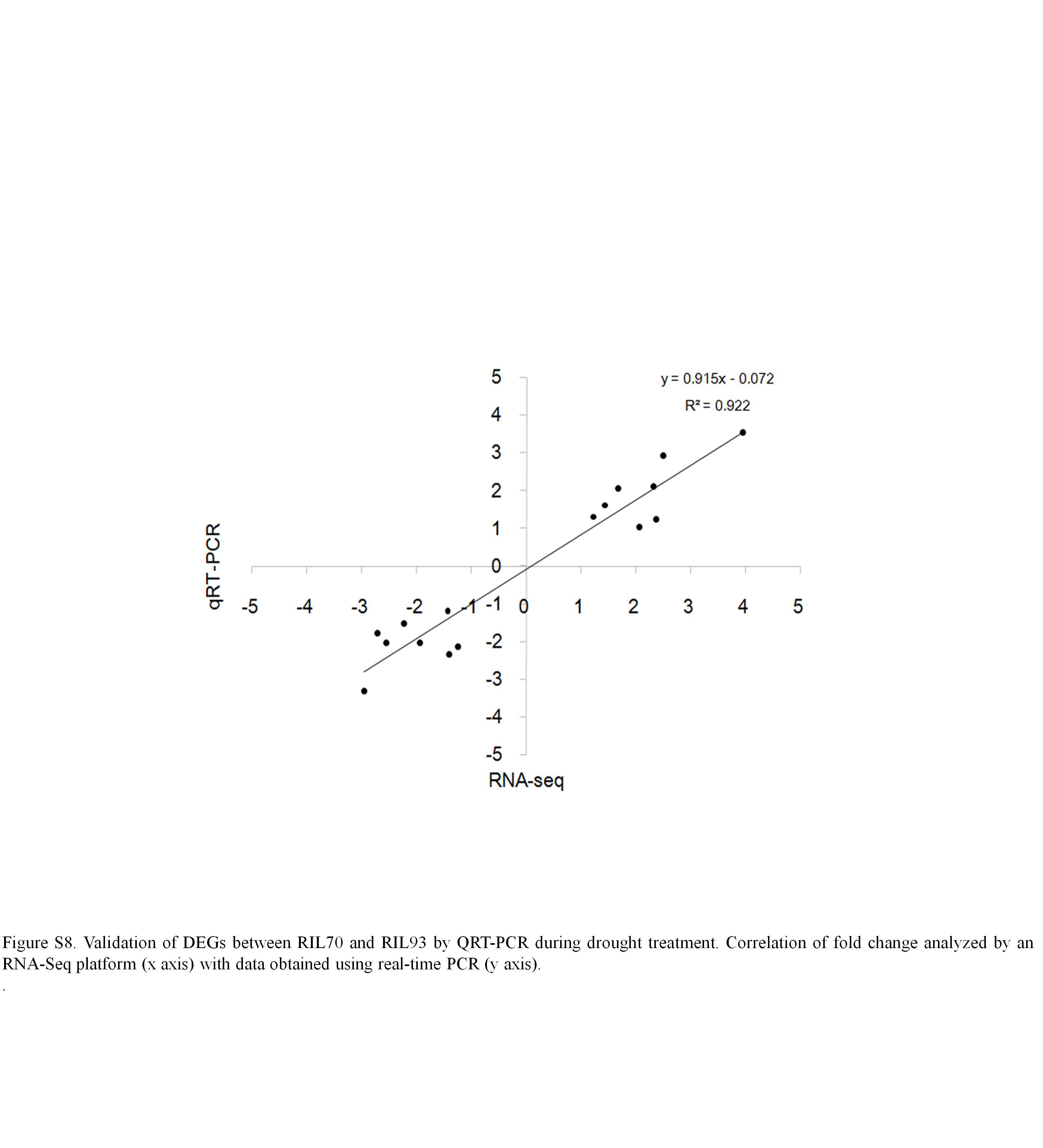

Supplement: Supplementary file 8 [file Image8.JPEG]

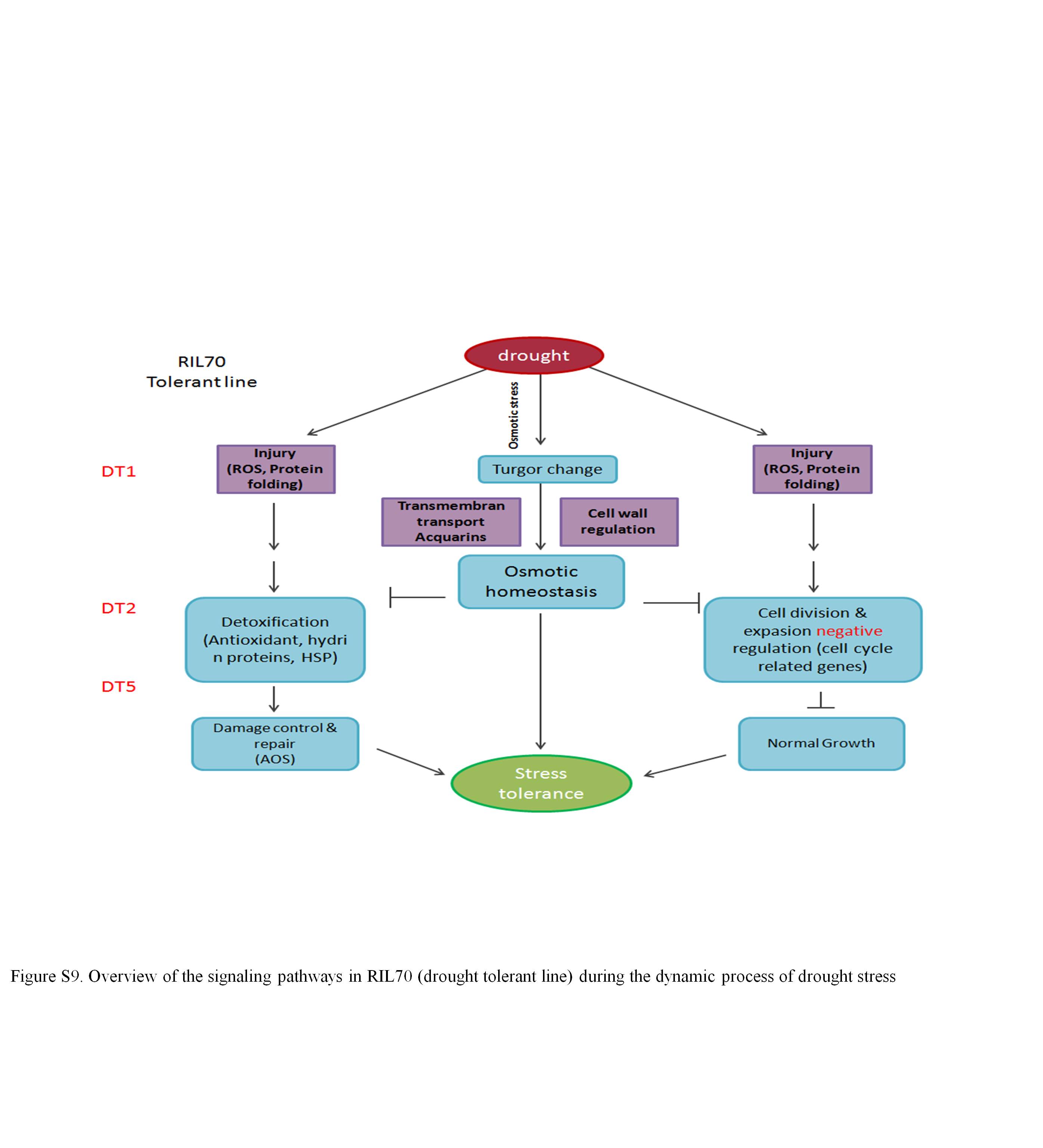

Supplement: Supplementary file 9 [file Image9.JPEG]
